# Direct growth of uniform carbon nitride layers with extended optical absorption towards efficient water-splitting photoanodes

**DOI:** 10.1038/s41467-020-18535-0

**Published:** 2020-09-17

**Authors:** Jiani Qin, Jesús Barrio, Guiming Peng, Jonathan Tzadikov, Liel Abisdris, Michael Volokh, Menny Shalom

**Affiliations:** Department of Chemistry and Ilse Katz Institute for Nanoscale Science and Technology, Ben-Gurion University of the Negev, Beer-Sheva, 8410501 Israel

**Keywords:** Solar fuels, Photocatalysis, Nanoscale materials

## Abstract

A general synthesis of carbon nitride (CN) films with extended optical absorption, excellent charge separation under illumination, and outstanding performance as a photoanode in water-splitting photoelectrochemical cells is reported. To this end, we introduced a universal method to rapidly grow CN monomers directly from a hot saturated solution on various substrates. Upon calcination, a highly uniform carbon nitride layer with tuned structural and photophysical properties and in intimate contact with the substrate is obtained. Detailed photoelectrochemical and structural studies reveal good photoresponse up to 600 nm, excellent hole extraction efficiency (up to 62%) and strong adhesion of the CN layer to the substrate. The best CN photoanode demonstrates a benchmark-setting photocurrent density of 353 µA cm^−2^ (51% faradaic efficiency for oxygen), and external quantum yield value above 12% at 450 nm at 1.23 V versus RHE in an alkaline solution, as well as low onset potential and good stability.

## Abstract (web summary)

Photoelectrochemical cells (PEC) can convert sunlight and water directly to a hydrogen fuel. Here a robust metal-free carbon nitride-based layer is used as an efficient photoanode for water-splitting PEC.

## Introduction

Carbon nitride (CN) materials have emerged as promising cheap and benign semiconductors for photoelectrochemical (PEC) cells during the last decade, owing to their stability under harsh conditions and suitable energy band edges for water-splitting and other chemical transformations^1–9^. However, the PEC performance of CN materials is still low compared to the state-of-art semiconductors^10–14^ and to their possible theoretical value. The CNs’ intrinsic moderate light-harvesting properties and poor charge separation properties alongside a limited variety of growth methods and monomers, which can be utilized to grow a continuous CN layer with an intimate contact to the substrate^4,15–18^ are obstacles to their successful PEC implementation. To date, several methods have been used to synthesize CN films, such as the doctor-blade technique, which require supramolecular assemblies as the precursor^19,20^, thermal vapor condensation^21–23^, solvothermal methods^24,25^, micro-contact printing^26^ and liquid-based methods^27–29^. However, despite the great progress achieved with the above-mentioned growth and deposition methods, most of them only permit the use of a restricted range of monomers as the starting precursor and some require more complicated conditions that are not suitable for all substrates and have scalability difficulties^30–32^. The small number of growth methods and usable monomers hinders the quest for CN materials with enhanced optical and electronic properties, which are mandatory characteristics for further CN-PEC improvement. Therefore, new deposition methods that allow the use of a wider range of monomers to cast multiple CN layers with diverse chemical, morphological, and photophysical properties are still highly needed to achieve the required substantial progress towards practical CN-based PECs. Moreover, to this day, most of the CN materials have only exhibited strong light response up to ~420 nm in PEC, because of their wide bandgap, which is only suitable for harvesting a small portion of the solar spectrum^33–36^. Therefore, increasing the photoresponse of CN layers in the visible region is crucial for further PEC activity enhancement.

Herein we demonstrate a simple and general method to grow porous CN films with extended optical absorption, improved charge separation properties under illumination, and intimate contact with the substrate via the direct and fast growth of monomers from saturated solutions, followed by calcination at high temperature. In addition, to improve the charge separation, connection to the substrate, and overall PEC properties, we introduce melamine vapor during the layer’s growth^37^. The best CN film, based on thiourea as a precursor, CN_TM_, exhibits a photoanodic photocurrent of 353 µA cm^−2^ at 1.23 V versus (vs.) reversible hydrogen electrode (RHE) in 0.1 M KOH aqueous solution under one-sun illumination, a low onset potential of 0.32 V, and a high incident photon-to-current conversion efficiency (IPCE) up to 600 nm. Notably, gas evolution rates of 1.88 µmol h^−1^ cm^−2^ for H_2_ and 0.91 µmol h^−1^ cm^−2^ for O_2_ are detected.

## Results

### Preparation and characterization of CN_T_ films

**Fig. 1:**
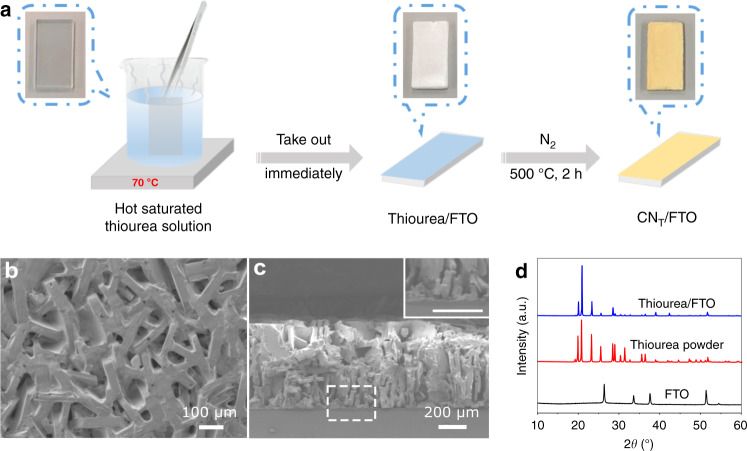
Carbon nitride film preparation from thiourea (CN_T_). **a** The proposed synthesis process of CN_T_ film on FTO (the corresponding physical photos are shown within the dotted line). **b** Top-view SEM image of the thiourea film on FTO. **c** Cross-sectional SEM image of a 3-layer thiourea film on FTO (inset: a magnified SEM image of the squared area). **d** XRD patterns of clean FTO, thiourea powder, and thiourea film on FTO.

The new synthetic path is illustrated in Fig. 1a. To deposit thiourea directly on fluorine-doped tin oxide (FTO)-coated glass, a clean FTO glass is immersed into a hot (70 °C) saturated thiourea aqueous solution (ca. 0.9 g mL^–1^) for 1 s and subsequently taken out, yielding a uniform film layer of thiourea on FTO after the remaining liquid is left to dry. The thickness of the thiourea film can be tuned by sequential dip-dry cycles. Top-view scanning electron microscopy (SEM) of the thiourea film reveals a cohesive and oriented network structure (Fig. 1b), which is strikingly different from that of the pristine thiourea powder (Supplementary Fig. 1) and indicative of a good adhesion after recrystallization. Cross-sectional SEM (Fig. 1c) shows a uniform thickness and close contact to FTO. X-ray diffraction (XRD) patterns of the thiourea film and the pristine thiourea powder show similar crystal structures, with differences only in the relative intensity of some of the peaks, a result of variations in the orientation of the crystals in the samples (Fig. 1d).

**Fig. 2:**
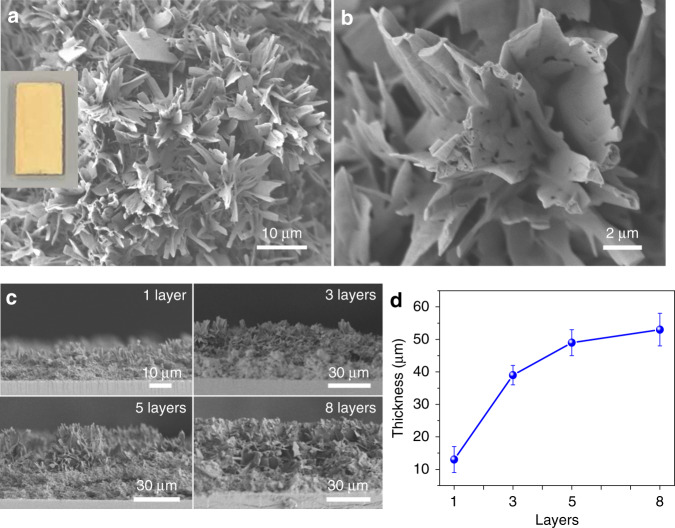
CN_T_ film thickness control. **a** Top-view SEM image (inset: digital image of CN_T_/FTO electrode) and **b** magnified top-view SEM image of CN_T_ film. **c** Cross-sectional SEM images of the different thicknesses CN_T_ films on FTO. **d** Relationship between the final thickness of CN_T_ films and the corresponding thiourea layers. Each data point represents an average of three different CN_T_ electrodes; error bars represent standard deviation.

The CN films were prepared by the calcination of the thiourea films at 500 °C for 2 h under nitrogen atmosphere (the proposed self-polymerization of thiourea into CN was given by Zhang et al.^38^); this resulted in uniform yellow-colored carbon nitride layers (CN_T_, T stands for thiourea) on FTO (Fig. 1a). A top-view SEM image of a CN_T_ electrode (Fig. 2a) presents a uniform and nanostructured architecture, which is dissimilar to the corresponding CN_T_ powder (Supplementary Fig. 2). High-magnification SEM (Fig. 2b) discloses a nanosheets structure with many pores, owing to the release of condensation products during calcination. The thickness of CN_T_ films (Fig. 2c) can be easily tuned from 13 to 53 μm (Fig. 2d) by adjusting the starting thickness of the thiourea layer (Supplementary Fig. 3).

All CN_T_ electrodes present chemical structural properties that are similar to those of CN_T_ powder (Supplementary Fig. 4), which indicates a successful CN layer synthesis. XRD patterns (Supplementary Fig. 4a) of all CN_T_ electrodes exhibit a strong and sharp peak at 27.4°, which is attributed to the (002) interlayer distance of graphitic CN. Another small reflection ca. 13° is attributed to the in-plane heptazine repeating units, i.e., (100)^33^. The Fourier-transform infrared (FTIR) spectra exhibit pronounced absorption peaks in the 1200–1600 cm^–1^ range (Supplementary Fig. 4b), which are assigned to the characteristic stretch modes of aromatic CN heterocycles. The peak at 810 cm^–1^ corresponds to the breathing mode of the heptazine units. The stretching vibration between 3000 and 3600 cm^–1^ indicates the existence of unreacted NH and/or NH_2_ groups^33,39^. The UV–Vis spectra of all the CN_T_ electrodes (Supplementary Fig. 4c) disclose a direct bandgap in the 2.15–2.38 eV range, with a long absorption tail that extends beyond 700 nm. Altering the layer thickness from 13 to 53 μm leads to a slight redshift of the optical edges, together with an increased optical density. In accordance with the absorption behavior, the photoluminescence (PL) spectra (Supplementary Fig. 4d) of all CN_T_ electrodes indicate a decrease of the PL intensity with increasing electrode thickness probably due to the contribution of defect states (bulk recombination) and possibly also increased reabsorption of emitted photons.

The chemical composition and oxidation state of the elements in the synthesized electrode were investigated by X-ray photoelectron spectroscopy (XPS), see Supplementary Fig. 5. The high-resolution spectrum of C 1*s* exhibits two peaks located at 284.6 and 287.9 eV, which belong to *sp*^2^ C–C bonds and N–C=N coordination, respectively. In the N 1*s* spectrum, three main peaks at 398.4, 399.9, and 401.0 eV were detected and assigned to *sp*^2^ C–N=C bonds, the tertiary nitrogen N–(C)_3_ groups, and the amino groups (C–NH–C and C–NH_2_), respectively, while a peak at 403.9 eV was assigned to charging effects^33,40^. In the O 1*s* spectrum, a peak at 532.1 eV, due to adsorbed water, was observed^41^. The high-resolution spectrum of S 2*p* shows the absence of sulfur in the final CN.

### PEC performance of the CN_T_ electrode

**Fig. 3:**
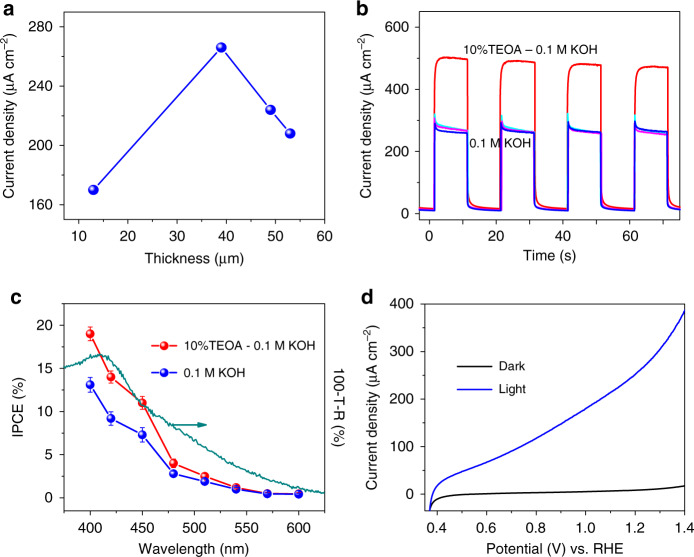
PEC characterization of CN_T_ electrodes. **a** Photocurrent–thickness relationship of CN_T_ electrodes. **b** Photocurrent density curves (chronoamperometry) of CN_T_ electrodes at 1.23 V vs. RHE in 0.1 M KOH (blue, cyan, and magenta lines) and in 0.1 M KOH containing 10% (v/v) TEOA (red line). **c** IPCE plots of CN_T_ electrode as a function of incident wavelength and UV–Vis spectrum of CN_T_ electrode. **d** LSV curves of CN_T_ electrode in 0.1 M KOH with and without light. All the light conditions are calibrated one-sun illumination. Each IPCE (%) data point represents an average of three different CN_T_ electrodes; error bars represent standard deviation.

PEC measurements in a three-electrode configuration under one-sun illumination reveal an optimal photoresponse for a CN_T_ layer thickness of 39 µm (Fig. 3a). The photocurrent density reaches 266 µA cm^–2^ at 1.23 V vs. RHE at this thickness. These results imply that there is a sufficient electron diffusion length within the CN_T_ electrode. However, the diffusion of electrons is not yet optimal as can be deduced from a front-side illumination measurement (Supplementary Fig. 6). Under front-side illumination, not all the photogenerated electrons reach the FTO glass due to the large distance that the electrons should travel. A further increase in the film’s thickness results in a decline in photocurrent, which probably stems from reduced light penetration within the electrode and increased electron–hole recombination within the active layer before they react^41^. In addition to the thickness, the influence of the calcination temperature during the preparation of the CN_T_ electrode on its morphology and PEC characteristics were also explored (Supplementary Figs. 7 and 8, respectively). As shown in Supplementary Fig. 8a, the CN_T_ electrode calcined at 500 °C exhibits the best PEC performance.

To determine the reproducibility of the CN_T_ electrode PEC performance, photocurrent experiments were performed on three CN_T_ electrodes, which were prepared in three separate synthetic batches. The photocurrent curves (Fig. 3b) of the three electrodes coincide very well (within ±10 µA cm^–2^), demonstrating the high repeatability of the new synthetic path of CN_T_ electrodes. The calculated incident photon-to-current conversion efficiency (IPCE) of CN_T_ electrodes at several illumination wavelengths (Fig. 3c) shows a direct correlation with light absorption. A high IPCE value of 13% is achieved at 400 nm, and a photoresponse can be detected up to 600 nm, which was seldom reported for unmodified CN electrodes. Linear sweep voltammetry (LSV) curves (Fig. 3d) of CN_T_ electrode in the dark and under one-sun illumination demonstrate a typical PEC behavior, with an onset potential at 0.38 V vs. RHE, and an increase in photocurrent at higher bias potentials.

To estimate the hole extraction efficiency, 10% (v/v) triethanolamine (TEOA), an efficient hole scavenger, was added to the system. The facile hole extraction leads to an increase in the photocurrent to 470 µA cm^−2^ (Fig. 3b). In good agreement with this result, the IPCE value at 400 nm is improved to 19% (Fig. 3c). It is assumed that most holes are successfully extracted from the layer to oxidize TEOA, essentially removing the kinetic barrier of the oxidation half-reaction, allowing a maximal photocurrent to be obtained. Based on this assumption, the calculated charge transfer efficiency of CN_T_ electrode at 1.23 V vs. RHE in 0.1 M KOH is 57 % (Supplementary Fig. 9), which is relatively high in the absence of any co-catalyst^17,31,42^. We note, that as we could not obtain a photocurrent plateau in the presence of TEOA, the actual values are probably lower.

### Growing CN_T_ films on different substrates

**Fig. 4:**
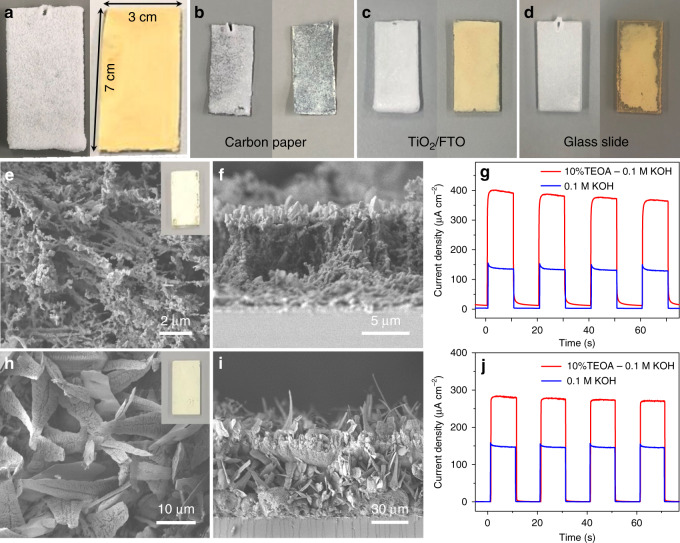
Characterization of different CN films. Digital images of thiourea film (left) and CN_T_ film (right) on different substrates: **a** Large-sized FTO, **b** carbon paper, **c** TiO_2_-coated electrode, and **d** glass slide. **e** Top-view SEM image (inset: digital image of CN_U_ electrode) and **f** cross-sectional SEM image of CN_U_ film on FTO. **g** Photocurrent density curves (chronoamperometry) of CN_U_ electrode at 1.23 V vs. RHE. **h** Top-view SEM image (inset: digital image of CN_D_ electrode) and **i** cross-sectional SEM image of CN_D_ film on FTO. **j** Photocurrent density curves (chronoamperometry) of CN_D_ electrode at 1.23 V vs. RHE.

To show the generality of this new method, CN_T_ film was grown on FTO with different sizes and shapes, and on other substrates, such as carbon paper, TiO_2_-coated electrode, and a glass slide. Figure 4a and Supplementary Fig. 10 show that regardless of the size and shape of the FTO substrate, both thiourea and the resulting CN films cover the entire FTO area well. On the other substrates, uniform and continuous CN_T_ films were also obtained (Fig. 4b–d). The corresponding SEM characterization demonstrates that the morphologies of CN_T_ films on different substrates are similar to those on FTO (Supplementary Figs. 11–13), suggesting a negligible effect of the substrates on the resulting CN_T_ film morphology.

### Synthesis of CN films using different precursors

The generality of the developed CN film fabrication method was further demonstrated by using different monomers. Taking urea (ca. 2.4 g mL^–1^ at 70 °C in water) as an example, we showed that a uniform and ordered urea film on FTO can be obtained by using this method (Supplementary Fig. 14). The method was then extended to monomers with lower solubility than thiourea and urea, such as dicyandiamide (DCDA, ca. 0.3 g mL^–1^ at 70 °C). Supplementary Figure 15 shows that only a partial formation of a DCDA film is obtained on FTO, due to its low solubility in water. With the addition of a preliminary seeding layer step, the formed DCDA is evenly spread on the entire FTO (Supplementary Fig. 16). After calcination, the obtained CN_U_ (CN synthesized from urea) electrode presents a loose coral-like morphology (Fig. 4e) while CN_D_ (CN synthesized from DCDA) looks like blades of grass (Fig. 4h). Both show a highly uniform microstructure, dissimilar to the corresponding precursor and CN powders (Supplementary Figs. 14c, 16e, and 17). Figure 4f, i demonstrate that the resulting CN_U_ and CN_D_ films are well attached to the FTO and present good PEC performance (Fig. 4g, j, and Supplementary Fig. 18). High photocurrents of 368 and 133 µA cm^−2^ were obtained from the CN_U_ electrode (Fig. 4g), and 277 and 145 µA cm^−2^ were achieved with the CN_D_ electrode (Fig. 4j), with or without a hole scavenger, respectively. Similarly, the two monomers can form porous and continuous CN films on other different substrates (Supplementary Figs. 19–21). In addition, two other monomers—ammonium thiocyanate (NH_4_SCN, abbreviated as NS, ca. 4.6 g mL^–1^ at 70 °C in water) and guanidine carbonate (abbreviated as GC, ca. 0.7 g mL^–1^ at 70 °C in water)—also allow uniform CN films formation by this method, leading to high PEC performance (Supplementary Figs. 22 and 23).

### Synthesis of CN_T_ films using other methods

For comparison, three other methods were used to synthesize CN photoelectrodes from thiourea. Direct calcination of thiourea powder spread on pristine FTO led to the formation of an uneven and cracked CN layer (Supplementary Fig. 24a). Drop-casting a slurry, followed by calcination at 300 °C for 1 h to increase adhesion gave a uniform CN film (Supplementary Fig. 24b). However, the layer peeled off upon contact with the electrolyte solution. A doctor-blade method, which is based on using thiourea as the precursor turned out to be infeasible owing to improper paste formation (Supplementary Fig. 24c).

### Characterization and PEC performance of the CN_TM_ electrode

**Fig. 5:**
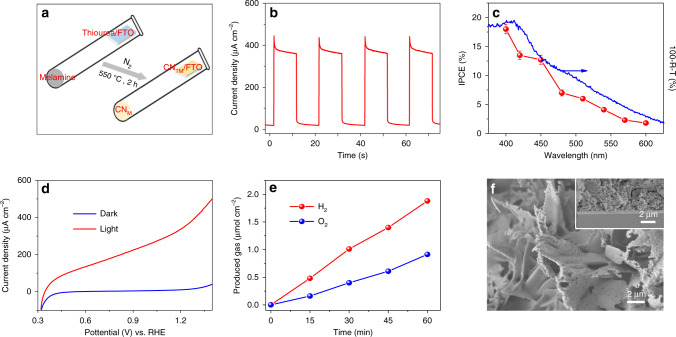
Characterization of CN_TM_ photoanodes. **a** Schematic synthetic process of CN_TM_ film on FTO. **b** Photocurrent density (chronoamperometry) upon 1-sun cycling illumination of CN_TM_ electrode at 1.23 V vs. RHE in 0.1 M KOH. **c** IPCE plots of CN_TM_ as a function of incident wavelength in 0.1 M KOH and the corresponding UV–Vis spectrum of the CN_TM_ electrode. **d** LSV curves of CN_TM_ electrode in 0.1 M KOH. **e** Time-production plot of the H_2_ and O_2_ generated from the PEC system with CN_TM_ at 1.23 V vs. RHE in 0.1 M KOH upon 1-sun illumination. **f** Top-view (inset: cross-sectional) SEM images of CN_TM_ film on FTO. (The light yellow powder labeled as CN_M_ in frame **a** refers to carbon nitride obtained by calcination of melamine powder at 550 °C for 2 h at the bottom of the tube). Each IPCE (%) data point represents an average of three different CN_TM_ electrodes; error bars represent standard deviation.

As the water-splitting reaction occurs on the photoelectrode’s surface, a surface modification could greatly affect the defect states and charge transfer to the electrolyte without varying the bulk properties of the photoelectrode^15,43,44^. To further improve the PEC performance of the discussed CN_T_ electrode, melamine powder was added to the bottom of the tube, and the thiourea/FTO was then placed close to the nozzle (Fig. 5a). After calcination, a slightly darker yellow CN film was obtained, named here as CN_TM_. The surface modification greatly improves the PEC performance of the CN; the modified CN_TM_ electrode calcined at 550 °C shows the best performance (Fig. 5b and Supplementary Fig. 25). The photocurrent and IPCE value at 400 nm reach up to 353 µA cm^−2^ and 18%, respectively (Fig. 5b, c). In addition, an IPCE above 12% was measured at 450 nm and up to 2% at 600 nm, indicating an improved visible light response. Chronoamperometric curves (Supplementary Fig. 26) of five different CN_TM_ electrodes match very well (within ±20 µA cm^–2^), indicating the good repeatability of the new synthetic path of CN_TM_ electrodes. Upon addition of 10% (v/v) TEOA hole scavenger to the system, the photocurrent increases to 565 µA cm^−2^ (Supplementary Fig. 27). This means an improved charge transfer efficiency of the CN_TM_ electrode in 0.1 M KOH, up to 62% (Supplementary Fig. 28) thanks to the surface modification. The improved charge separation is also evident in the lower onset potential of the CN_TM_ electrode (0.32 V vs. RHE) than CN_T_ electrode (0.38 V), indicating that a non-zero photoresponse is generated at almost 1 V below the potential of water oxidation (Fig. 5d). We note that these PEC values are among the best-performing for CN materials in the absence of a hole scavenger (Supplementary Table 1). Based on the good performance of CN_TM_ photoanodes, the gas evolution during the reaction was detected by gas chromatography (Supplementary Fig. 29a), which showed a nearly 2:1 molar ratio of H_2_ and O_2_ generation rate, about 1.88 and 0.91 µmol h^−1^ cm^−2^, respectively (Fig. 5e), with calculated faradaic efficiency (FE) of 53 ± 1% for H_2_ and 51 ± 1.5% for O_2_. After a 1-h PEC reaction, about 50% of the initial photocurrent is preserved (Supplementary Fig. 29b). Post-characterization of the CN_TM_ electrode (Supplementary Fig. 30) shows that the crystallinity of the CN_TM_ electrode becomes slightly weaker and a small peak appears at 288.7 eV in the C 1*s* XPS spectrum, which is assigned to the C–O bond^45,46^, suggesting partial oxidation of the CN_TM_ electrode occurred during the 1-h reaction. This oxidation is further observed in the O 1*s* spectrum (Supplementary Fig. 30d), where a signal from N–C–O species appears at 531.2 eV^47^. Furthermore, we characterized the CN-layer after 12 h stability measurement to confirm the structural changes. Therefore, the decrease in photocurrent should be attributed to the partial oxidation of the CN photoanode and the weakening of crystallinity during the reaction in 0.1 M KOH.

Generally, water-spitting under neutral conditions is appealing. Thus, we have also measured the performance of CN_TM_ photoanodes in 0.1 M Na_2_SO_4_ (pH = 7.02). Supplementary Fig. 31a shows that it also exhibited an outstanding PEC performance with a photocurrent of 305 µA cm^−2^ at 1.23 V vs. RHE under one-sun illumination. Meanwhile, the CN_TM_ electrode is more stable under such conditions and higher rates of gas evolution (H_2_ and O_2_) were obtained relative to alkaline conditions (0.1 M KOH). Supplementary Fig. 31b shows there is almost no loss of photocurrent during the first 30 min. After an hour, the remaining photocurrent is about 72% of the initial photocurrent. The detected H_2_ and O_2_ were 2.26 and 1.08 µmol h^−1^ cm^−2^ (Supplementary Fig. 31c–d), respectively, corresponding to FE of 55% for H_2_ and 52% for O_2_.

Despite the introduction of melamine, the chemical structure, composition, as well as the morphology of the synthesized CN_TM_ film remains similar to CN_T_ with slight surface modification of the nanosheets (Fig. 5f and Supplementary Figs. 32–34). In addition, a small redshift of the direct optical bandgap from 2.33 to 2.26 eV is observed (Fig. 5c and Supplementary Fig. 35a). Combined with the results of Mott-Schottky measurements (Supplementary Fig. 35b, c), the conduction band and valence band positions of CN_TM_ electrode are –1.21 and 1.05 eV vs. Ag/AgCl, respectively (see scheme in Supplementary Fig. 35d), which thermodynamically allow both water reduction and oxidation. Moreover, electrochemical active surface area (ECSA) measurements (Supplementary Fig. 36) demonstrate that the ECSA of the CN_TM_ electrode is higher than that of the CN_T_ electrode, which is in line with the calculated Brunauer–Emmet–Teller (BET) specific surface area from N_2_ sorption experiments (9.2 m^2^ g^–1^ for CN_TM_ vs. 6.1 m^2^ g^–1^ for CN_T_, Supplementary Fig. 37). The ECSA and BET analysis suggest that the CN_TM_ layer has more accessible sites for PEC reactions. In addition, electrochemical impedance spectroscopy (EIS) measurements (Supplementary Figs. 8 and 38) reveal that the introduction of melamine decreases the charge transfer resistance of holes from the CN layer to the solution.

### Adhesion test

On the other hand, the addition of melamine during the synthesis increases the pressure inside the tube, which results in better contact between the CN and the FTO substrate (Fig. 5f, inset). We note that melamine can be replaced with another monomer (e.g., NH_4_SCN, who’s photoresponse is shown in Supplementary Fig. 39) that produces significant vapor pressure, leading to enhanced PEC performance. The latter accentuates the importance of the induced pressure during the film synthesis over the final performance. To confirm the strong adhesion, the prepared CN_TM_ electrode was subjected to ultrasonication and adhesive tape testing. After 5 min of ultrasonication, only slight turbidity is detected in the liquid (Supplementary Fig. 40), which mainly resulted from CN powder at the surface of the photoactive CN film. Additional ultrasonication for a total of 30 min did not increase the turbidity and the CN film remained unchanged. The tape adhesion experiment was then carried out on the same CN_TM_ electrode (after sonication, Supplementary Fig. 41). The CN_TM_ film remained intact even after an adhesive tape was pasted onto the film under pressure for 2 h. A control experiment with CN_T_ electrode (Supplementary Fig. 42a) shows that the CN_T_ solution is more turbid than the CN_TM_ one after 5 min of ultrasonication. It was clearly observed that with increasing ultrasonication time, the edges of the CN_T_ film are slowly falling off. This shows that the adhesion between CN_TM_ film and FTO is stronger than that of the CN_T_ film. The better contact between the FTO and CN_TM_ layer leads to lower resistance, which in turn enables the higher photocurrent and better stability. However, we note that most of the CN_T_ film remains intact after 30 min of ultrasonication and a subsequent tape-test (Supplementary Fig. 42b), indicating that it is still robust.

## Discussion

The reported simple, scalable, and versatile method allows the deposition of uniform carbon nitride layers with enhanced optical absorption and charge separation under illumination on various substrates. This deposition method relies on the fast growth of CN monomers directly on a substrate, followed by calcination at high temperature. Furthermore, we demonstrate that the introduction of melamine vapor during the calcination step significantly enhances the PEC activity of the CN electrodes thanks to surface modification. Detailed PEC studies unveil excellent hole extraction efficiency, up to 62%, together with good photoresponse over the visible range up to 600 nm (IPCE > 12% at 450 nm). The best CN film, based on thiourea as the starting monomer, exhibits a high photocurrent density of 353 µA cm^−2^, with FE of 53 ± 1% for H_2_ and 51 ± 1.5% for O_2_, and an IPCE value of 18% at 400 nm at 1.23 V vs. RHE in alkaline (0.1 M KOH) aqueous solution without sacrificial agents, providing a benchmark for CN photoanode materials.

## Methods

### Chemicals

All the chemical reagents were used without further purification. Thiourea (99%) and guanidine carbonate (GC) (99%) from Acros Organics. Urea (99%), dicyandiamide (DCDA) (99%), and melamine (99%) from Sigma-Aldrich. NH_4_SCN (NS) (98%) from Alfa Aesar. KOH (85%) and Na_2_SO_4_ (99%) from Loba-Chemie. Methanol (≥99.8%), ethanol (≥99%), and acetone (≥99%) from Bio-Lab Ltd. Triethanolamine (>99%) from Carl Roth. Fluorine-doped tin oxide (FTO)-coated glass (12–14 Ω sq^−1^) was purchased from Xop Glass company, Spain. Before using, the FTOs were thoroughly washed with an aqueous detergent (Alconox) solution, acetone, and ethanol in sequence and dried at 60 °C. Deionized water (18.2 MΩ cm resistivity at 25 °C, purified using a Millipore Direct-Q3 system) was used as the solvent for all the reported experiments.

### Characterization

A PANalytical’s Empyrean diffractometer was employed to conduct X-ray diffraction (XRD) measurements. Fourier transformed infrared (FTIR) spectra were collected on a Thermo Scientific Nicolet iN 10Mx infrared microscope. UV–Vis spectra were recorded on a Cary 100 spectrophotometer equipped with a diffuse reflectance accessory (DRA). An Edinburgh FI/FSTCSPC 920 fluorimeter was used to collect photoluminescence (PL) spectra. The morphologies of the samples were characterized using a scanning electron microscope (SEM, JEOL JSM-7400F equipped with a FEG source) operated at 3.5 kV using a secondary electrons detector. X-ray photoelectron spectroscopy (XPS) measurements were conducted on a Thermo Fisher Scientific ESCALAB 250 using monochromated Kα X-rays (1486.6 eV), and all the binding energies obtained in XPS spectra were calibrated using the C 1*s* peak at 284.6 eV. N_2_ (99.999%) adsorption-desorption measurements were performed using a Quantachrome autosorb IQ2 at 77 K. The specific surface area was calculated using the Brunauer–Emmet–Teller (BET) model. Pore‐size distribution was calculated using non‐localized density functional theory (NLDFT) from the nitrogen sorption measurements. The amount of generated gases in the cell of the PEC set up were analyzed using a gas chromatograph (Agilent 7820 GC system) equipped with a thermal conductivity detector (TCD).

### Material preparation

Thiourea/FTO: To deposit a thiourea film onto FTO, a clean FTO plate was immersed into a hot (70 °C) saturated thiourea aqueous solution and subsequently taken out. After the surface liquid was dried, a uniform film layer of thiourea on FTO was obtained. This step can be repeated several times to get different layers of thiourea film on FTO. Finally, the thiourea/FTO samples were dried in a 60 °C oven for subsequent use. Thiourea films on other substrates were obtained similarly.

Urea/FTO, NS/FTO, and GC/FTO: The preparing procedure of urea/FTO, NS/FTO, and GC/FTO is similar to that of thiourea/FTO, i.e., using urea, NH_4_SCN, and guanidine carbonate instead of thiourea, respectively. Urea on other substrates was also prepared using the same method.

DCDA/FTO: First, a clean FTO was dipped into a saturated DCDA solution in methanol at room temperature and was dried under air to form DCDA seeds. Secondly, the DCDA seeds/FTO was immersed into a hot (70 °C) saturated DCDA aqueous solution and subsequently taken out. A uniform layer of DCDA on FTO was thus obtained and dried at 60 °C. DCDA on other substrates was prepared using the same method.

CN_T_/FTO: To obtain a carbon nitride film on FTO, thiourea/FTO substrates were encapsulated in a glass tube filled with nitrogen. The glass tube was inserted into a ceramic tube furnace with N_2_ atmosphere. The oven was heated at a rate of 5 °C min^−1^ to 500 °C, when a calcination step at 500 °C for 2 h took place, resulting in a uniform yellow CN_T_ layer on FTO.

CN_U_/FTO, CN_D_/FTO, CN_NS_/FTO, and CN_GC_/FTO: CN_U_/FTO, CN_NS_/FTO, and CN_GC_/FTO were obtained by calcination of the corresponding precursor films at 500 °C for 2 h under N_2_. CN_D_/FTO was obtained by calcination of DCDA/FTO at 500 °C for 4 h under N_2_.

Carbon nitride (CN_T_, CN_U_, and CN_D_) films on other substrates were similarly obtained by changing the substrate on which the precursor deposition occurred.

CN_TM_/FTO: First, 1.0 g of melamine powder was added to the bottom of the tube, then the thiourea/FTO was placed close to the nozzle. The tube was then purged with N_2_, closed, and placed into an N_2_ oven. After calcination at 550 °C for 2 h, the CN_TM_/FTO was finally obtained. CN_TNS_/FTO was similarly obtained by using NH_4_SCN powder instead of the melamine powder.

The dimensions of all the reported substrates (FTO, carbon paper, a glass slide) were 1.2 cm × 2.5 cm unless otherwise specified.

### Photoelectrochemical analysis

A Newport 300 W Xe arc lamp equipped with air mass AM 1.5G and water filters was used as a photoexcitation light source. The intensity of incident light simulated as one-sun illumination was calibrated using a Newport 919P thermopile detector. The electrochemical analysis measurements were conducted in a conventional three-electrode cell on an Autolab potentiostat (Metrohm, PGSTAT302N) system, using a 1 cm^2^ Pt foil electrode as the counter electrode and an Ag/AgCl electrode as the reference electrode, respectively. 0.1 M KOH aqueous solution (pH = 13) and 0.1 M Na_2_SO_4_ aqueous solution (pH = 7.02) were used as the electrolyte and were purged with argon to remove O_2_ before the electrochemical measurements. All the potentials were converted to reversible hydrogen electrode (RHE) values using the Nernst equation at room temperature:

1 $$V_{{\mathrm{RHE}}} = V_{{\mathrm{Ag}}/{\mathrm{AgCl}}} + 0.059 \times {\mathrm{pH}} + 0.197.$$

Photocurrent measurements were conducted at 1.23 V vs. RHE and all photocurrents in the text and supporting materials were measured after three minutes of equilibration time unless otherwise specified. Mott-Schottky measurements were carried out in 1 M Na_2_SO_4_ aqueous solution using the same equipment.

Incident photon-to-current conversion efficiency (IPCE) values were calculated using:

2 $${\mathrm{IPCE}}\left( {\mathrm{\% }} \right) = \frac{{J\left( {{\mathrm{A}}\,{\mathrm{cm}}^{ - 2}} \right)\cdot 1240}}{{\lambda \left( {{\mathrm{nm}}} \right)\cdot I({\mathrm{W}}\,{\mathrm{cm}}^{ - 2})}}\cdot 100\%,$$

where *J* is the photocurrent density; *λ* is the wavelength of the incident monochromic light, which is controlled by different optical bandpass filters (Newport 10BBPF10 series) of 400, 420, 450, 480, 510, 540, 570, and 600 nm; *I* is the incident light power.

Hole extraction efficiency was calculated using:

3 $$\eta \left( {\mathrm{\% }} \right) = \frac{{J_{{\mathrm{KOH}}}}}{{J_{{\mathrm{TEOA}}}}}\cdot 100\%.$$

It is assumed that the extraction rate of photogenerated holes in the system is 100% after the addition of a hole scavenger (10% v/v TEOA). *J*_KOH_ is the photocurrent density obtained in 0.1 M KOH aqueous solution, while *J*_TEOA_ is the photocurrent density obtained in 0.1 M KOH containing 10% (v/v) TEOA.

The faradaic efficiency (FE) was calculated using:

4 $${\mathrm{FE}}({\mathrm{\% }}) = \frac{{m\cdot n\cdot F}}{{I\cdot t}}\cdot 100{\mathrm{\% }},$$

where *m* is the number of moles of gas produced; *n* is the number of reaction electrons; *F* is the Faraday constant; *I* is the photocurrent; *t* is the reaction time.

## Supplementary information

Supplementary information

## Data Availability

The data supporting the findings of this study are available from the corresponding author upon reasonable request.
