# Supplementary material for: Direct growth of uniform carbon nitride layers with extended optical absorption towards efficient water-splitting photoanodes
Source: Nat Commun. 2020 Sep 17;11:4701. doi: 10.1038/s41467-020-18535-0 (PMC7499157; doi:10.1038/s41467-020-18535-0)
Supplement: Supplementary file 1 — Supplementary information [file 41467_2020_18535_MOESM1_ESM.pdf]

Supplementary Information

Direct growth of uniform carbon nitride layers with extended optical absorption towards efficient water-splitting photoanodes

Qin *et al.*

Supplementary Figures

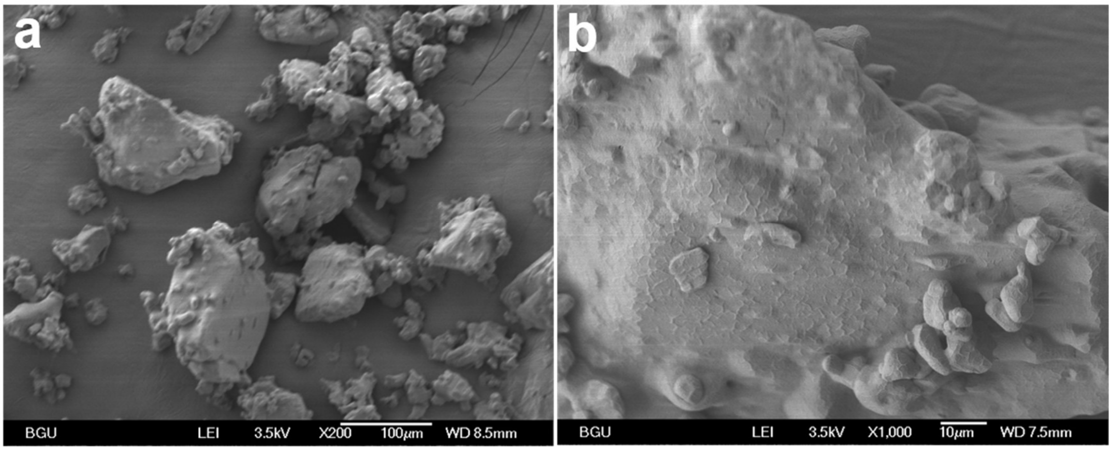

Supplementary Figure 1. SEM images of thiourea powder. a Low magnification. **b** High magnification.

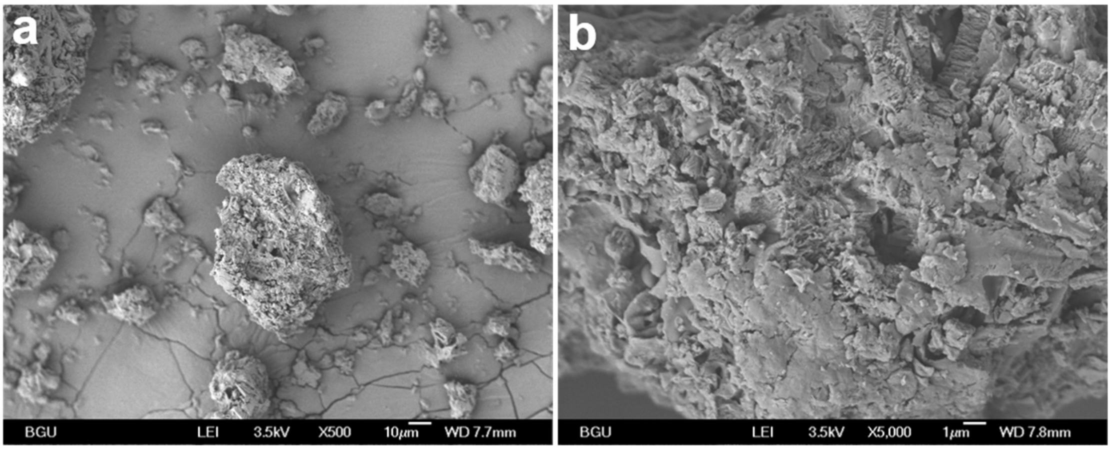

Supplementary Figure 2. SEM images of CN_T powder. **a** Low magnification. **b** High magnification.

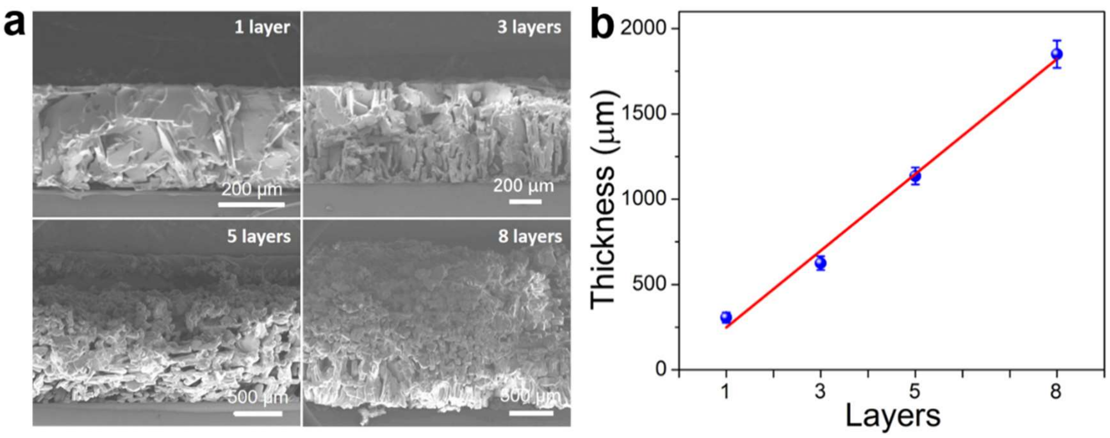

Supplementary Figure 3. The thickness of different layers thiourea film on FTO. a Cross-sectional SEM images of thiourea films with different number of layers (1, 3, 5, and 8) on FTO. **b** The relationship between the thiourea film thickness and the number of layers. Each thickness value (blue dots) is the average of three thiourea films on FTO. The error bars show standard deviation. The red line is a linear fit.

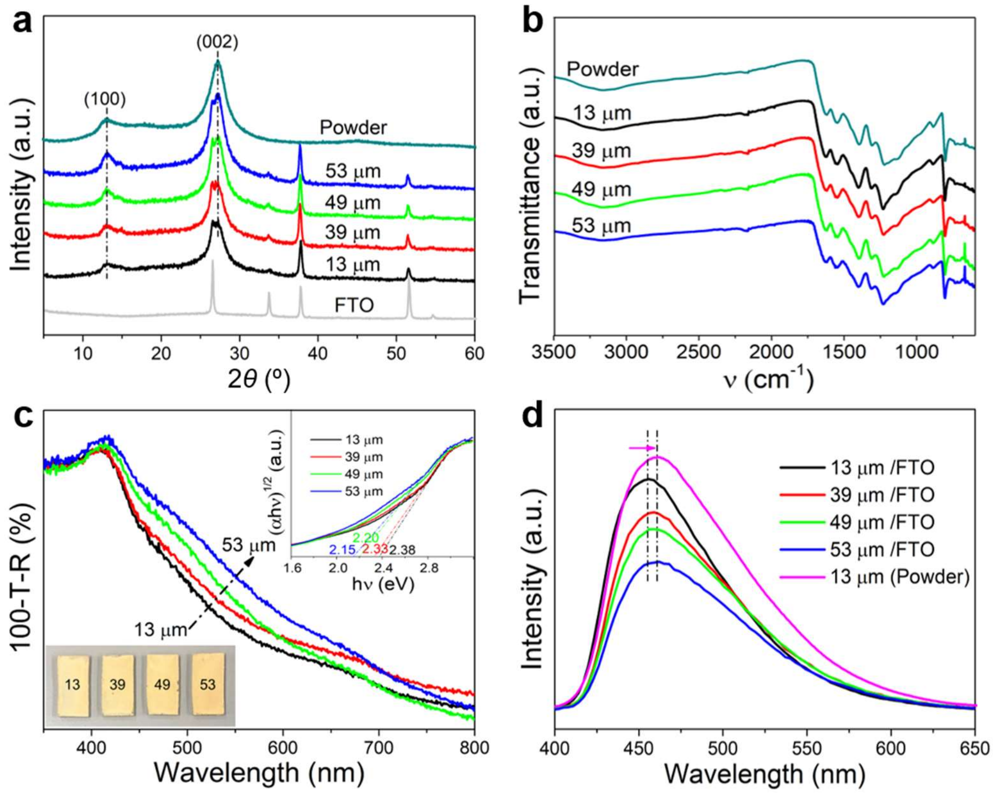

Supplementary Figure 4. Characterization of CN_T electrodes. **a** XRD patterns and **b** FTIR spectra of CN_T electrodes with different thickness and CN_T powder. **c** UV-vis spectra (inset: a digital photo of CN_T electrodes of different film thickness) and **d** PL spectra (excitation wavelength is λ_{ex} = 370 nm) of CN_T electrodes with different thickness (the magenta spectrum: CN_T powder scraped from the FTO substrate (13 μm/FTO)).

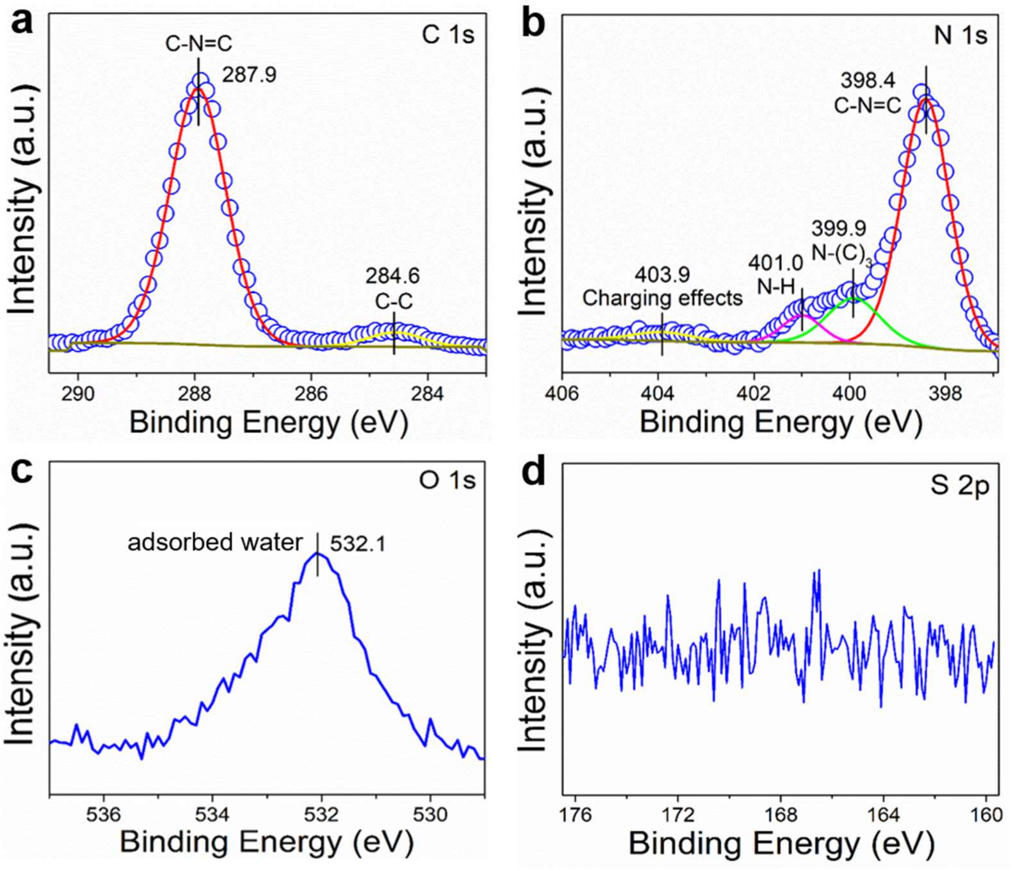

Supplementary Figure 5. XPS spectra from the CN_T electrode. a C 1s, **b** N 1s, **c** O 1s, and **d** S 2p.

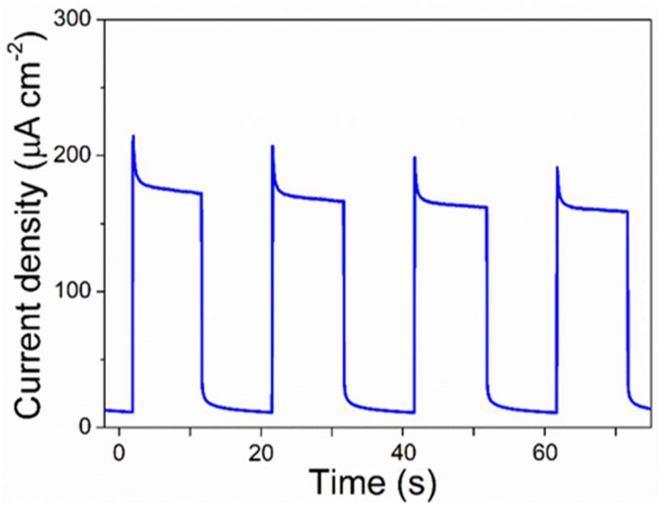

Supplementary Figure 6. Current density *vs.* time (chronoamperometry) of the CN_T electrode.

Measured at 1.23 V *vs.* RHE in 0.1 M KOH upon 1-sun cycling illumination from the front side.

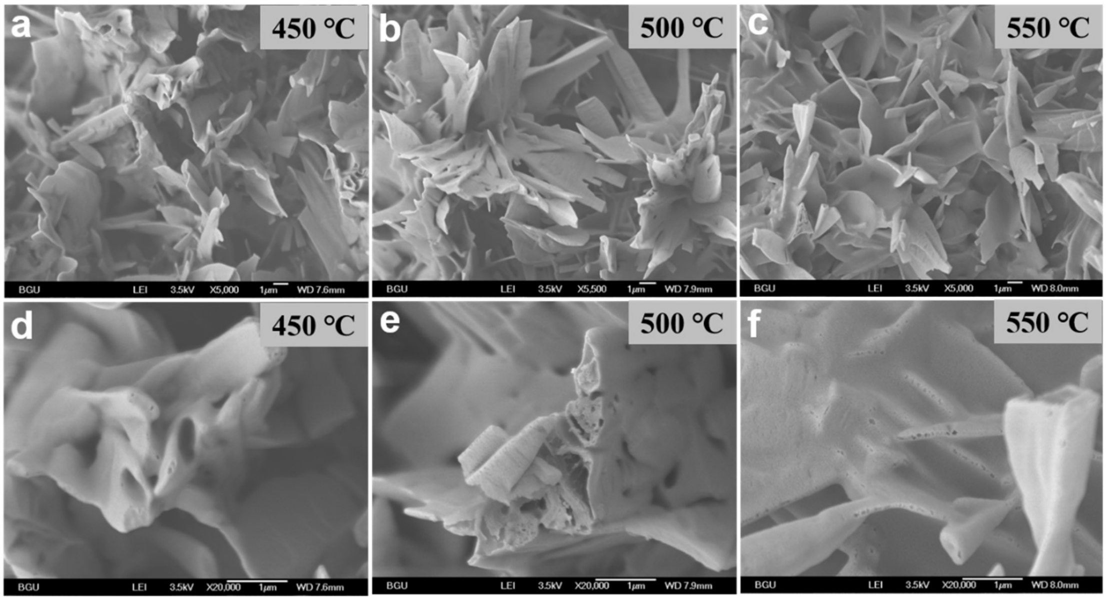

Supplementary Figure 7. SEM images of CN_T electrodes calcined at different temperatures. (**a, d**) 450 °C, (**b, e**) 500 °C, and (**c, f**) 550 °C. The SEM characterization reveals that CN_T electrodes calcined at different temperatures show similar morphology. Second row of images (**d**, **e**, and **f**) are higher-magnification of the first row (**a**, **b**, and **c**, respectively).

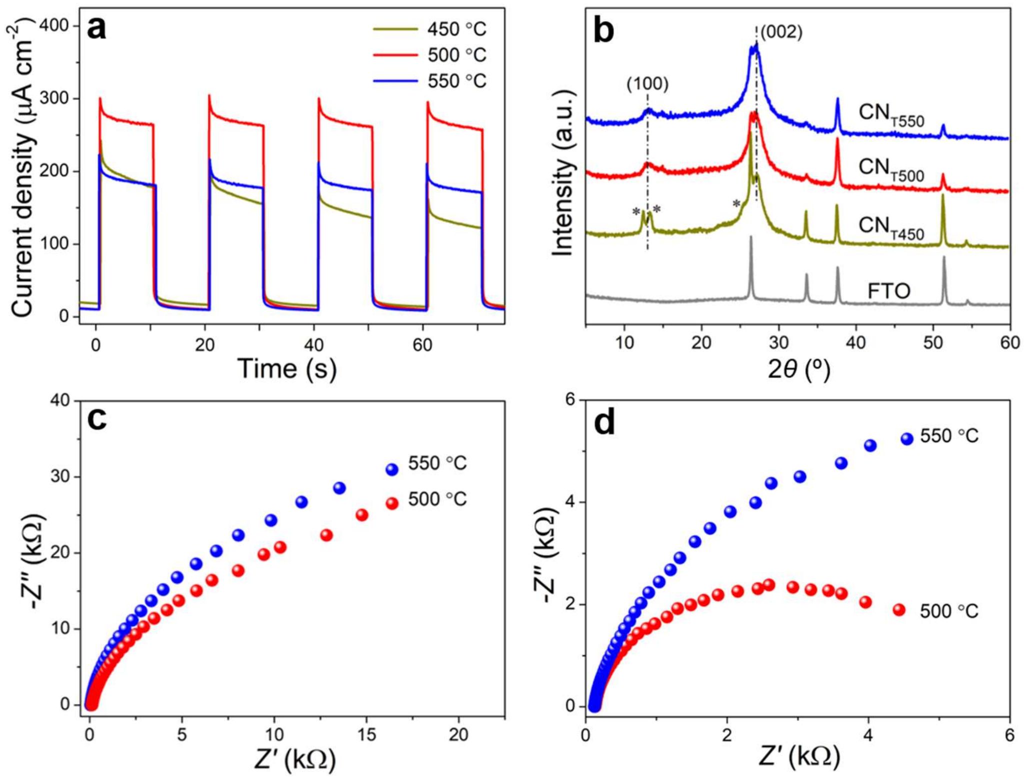

Supplementary Figure 8. Characterization of CN$_T$ electrodes obtained at different calcination temperatures. a Current density (chronoamperometry) at 1.23 V *vs.* RHE in 0.1 M KOH upon 1-sun cycling illumination. **b** XRD patterns. **c** Nyquist plots at 1.23 V *vs.* RHE under dark condition, and **d** Nyquist plots at 1.23 V *vs.* RHE upon 1-sun illumination. See further discussion in Supplementary Note 1.

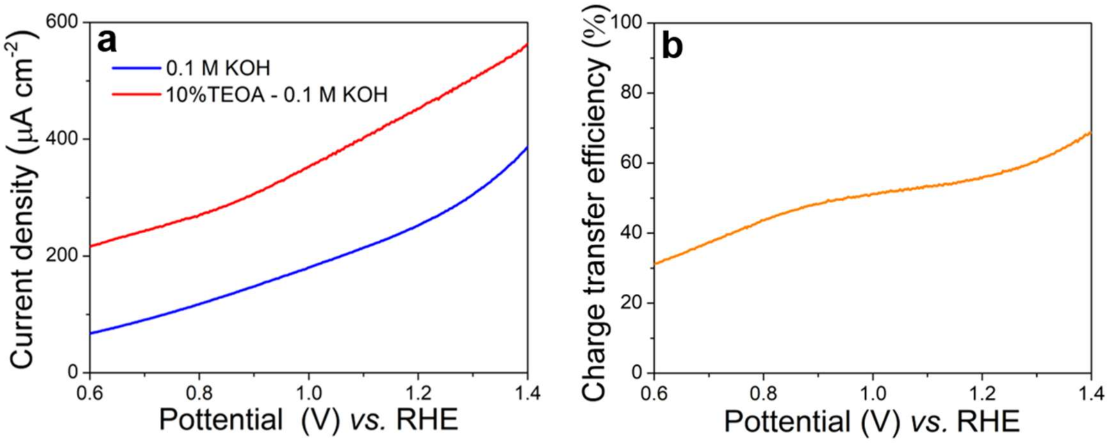

Supplementary Figure 9. Charge transfer efficiency of CN_T electrode. a Linear sweep voltammetry (LSV) curves of the CN_T electrode in 0.1 M KOH aqueous solution and in 0.1 M KOH containing 10% (v/v) TEOA upon 1-sun illumination. **b** The charge transfer efficiency plot of the CN_T electrode. The charge transfer efficiency being defined as the percentage of the photocurrent obtained in 0.1 M KOH relative to the one obtained in 0.1 M KOH with 10% v/v TEOA, assuming that all holes are successfully extracted from the layer to oxidize TEOA. This is an upper-limit calculation as we note that not all holes are successfully extracted under these conditions.

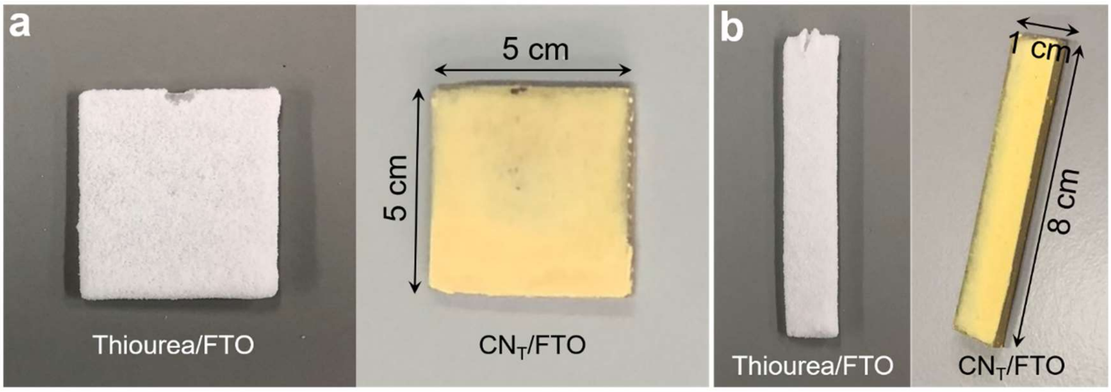

Supplementary Figure 10. Films deposited on large-sized FTO substrates. **a** 5 cm × 5 cm and **b** 1 cm × 8 cm. In each panel on the left (white film) is thiourea and on the right (yellow film) is CN_T.

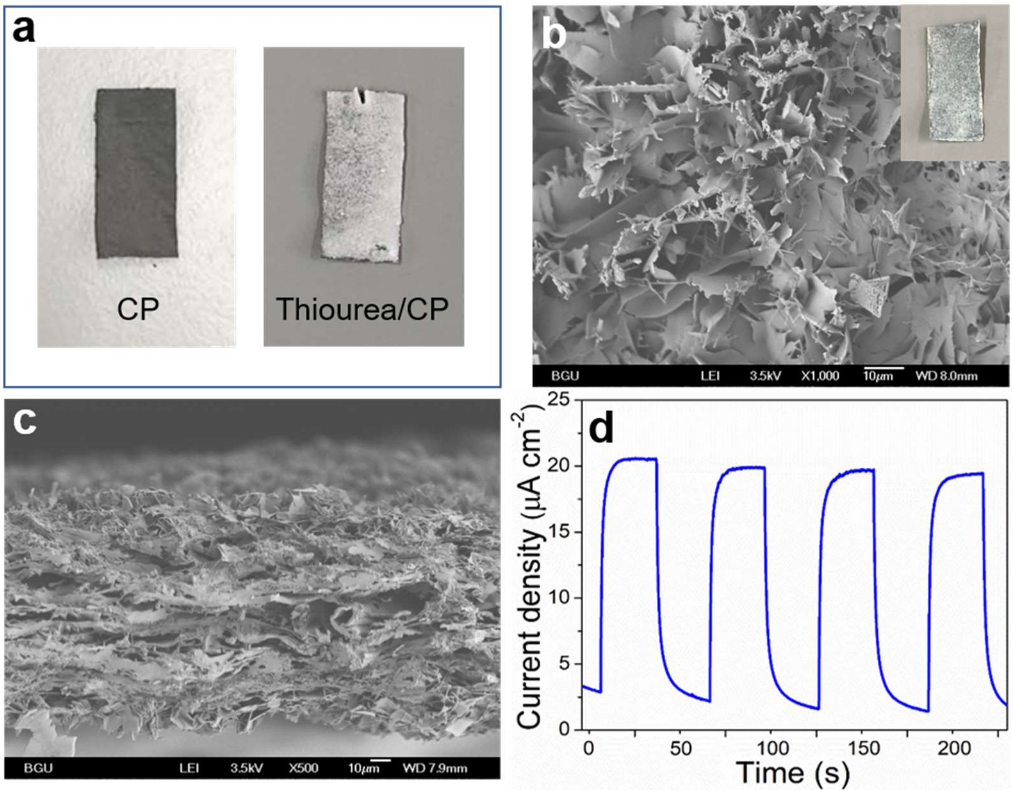

Supplementary Figure 11. CN_T film on carbon paper (CP). a Digital photos of CP (left) and thiourea film on CP (right). **b** Top-view and **c** cross-sectional SEM images of CN_T film on CP. **d** Current density (chronoamperometry) of CN_T/CP electrode at 1.23 V *vs.* RHE in 0.1 M KOH upon 1-sun cycling illumination.

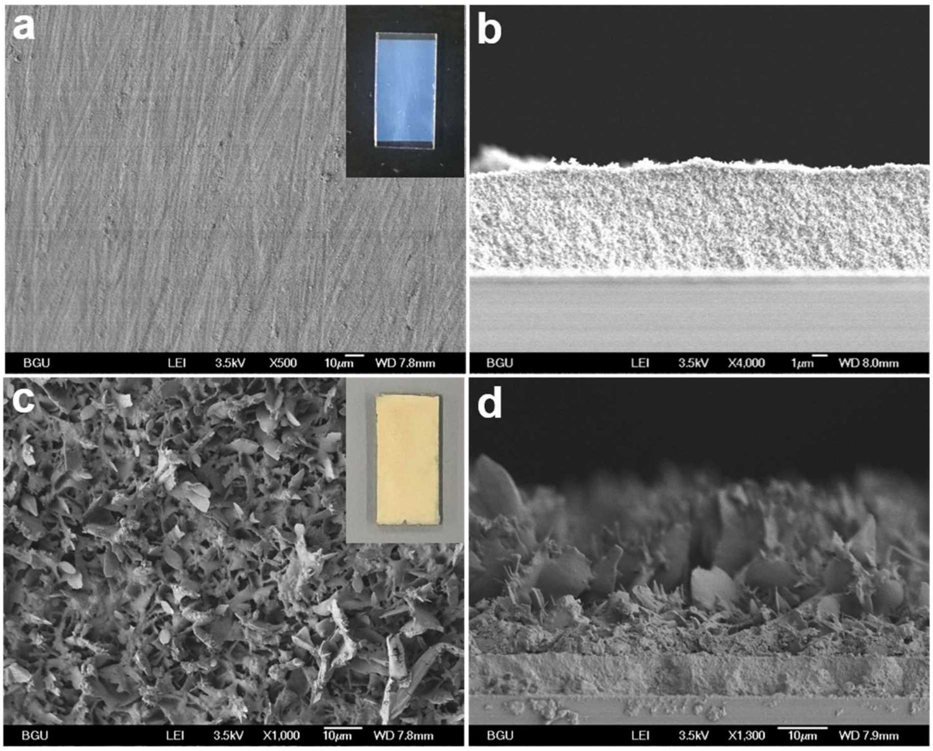

Supplementary Figure 12. CN_T film on TiO_2-coated FTO. a Top-view (inset: a digital photo) and **b** Cross-sectional SEM images of TiO_2-coated FTO. **c** Top-view, and **d** cross-sectional SEM images of CN_T film on TiO_2-coated FTO.

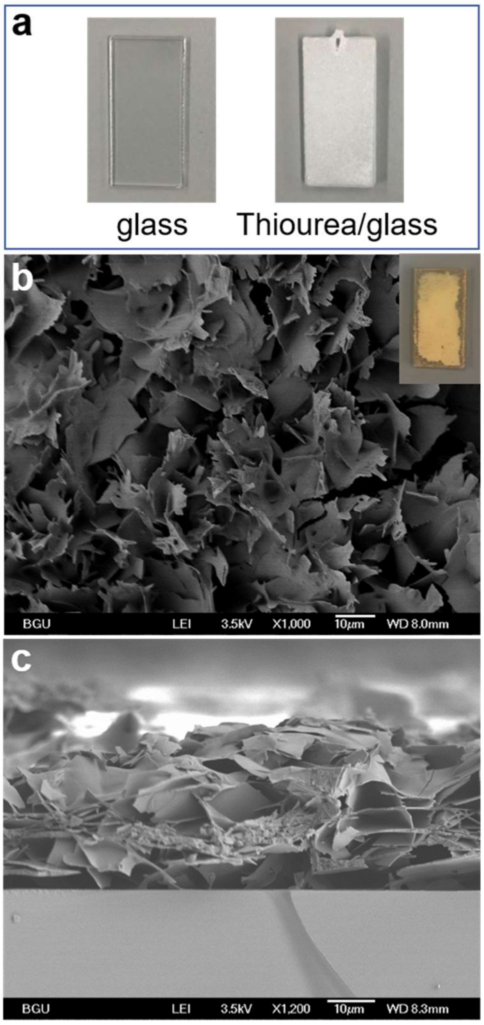

Supplementary Figure 13. CN_T film on glass slide. a Digital photos of a glass slide (before) and thiourea film on a glass slide (after deposition). **b** Top-view and **c** cross-sectional SEM images of CN_T film on glass slide.

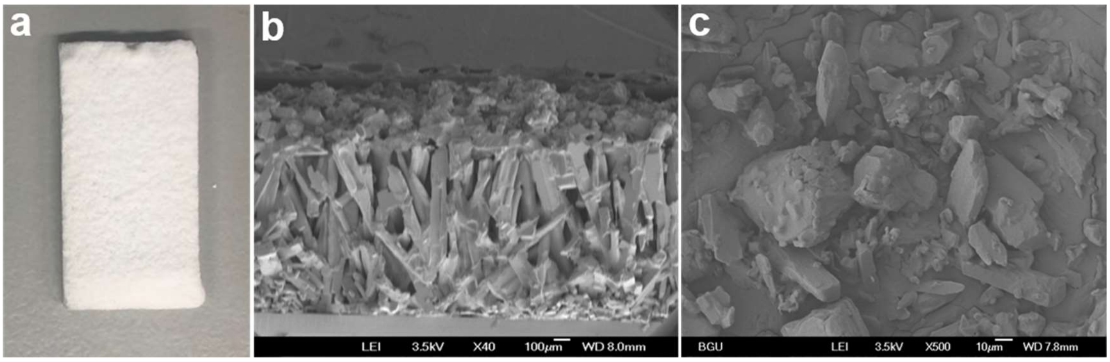

Supplementary Figure 14. Urea film on FTO. a digital photo and **b** cross-sectional SEM image of urea film on FTO. **c** SEM image of urea powder.

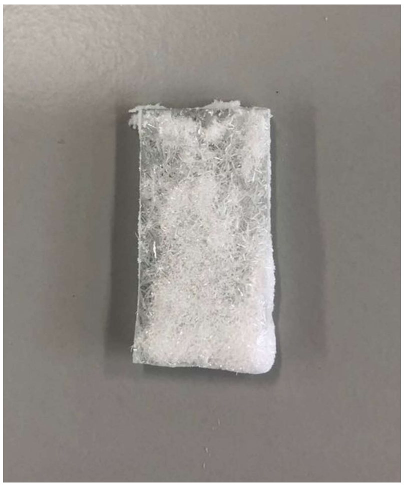

Supplementary Figure 15. A digital photo of DCDA film on FTO. This film was prepared by directly immersing a clean FTO-coated glass into a hot (70 °C) saturated DCDA aqueous solution.

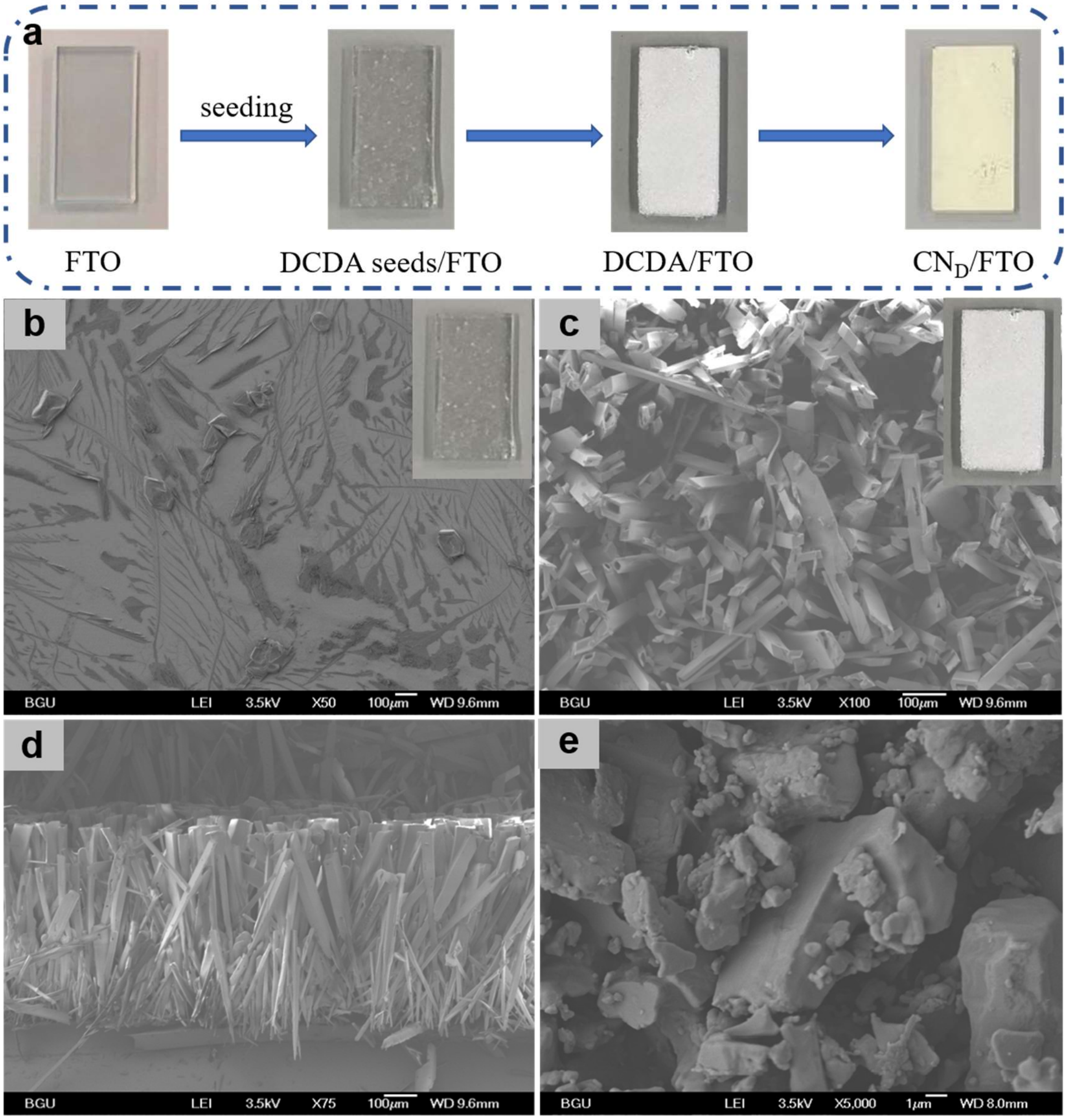

Supplementary Figure 16. DCDA film on FTO. a The proposed synthesis process of CN_D film on FTO, including a seeding step. **b** Top-view SEM image of DCDA-seeded FTO surface (inset: a digital photo of an FTO coated with DCDA crystal seeds). **c** Top-view and **d** cross-sectional SEM images of DCDA film on FTO. **e** SEM image of DCDA powder.

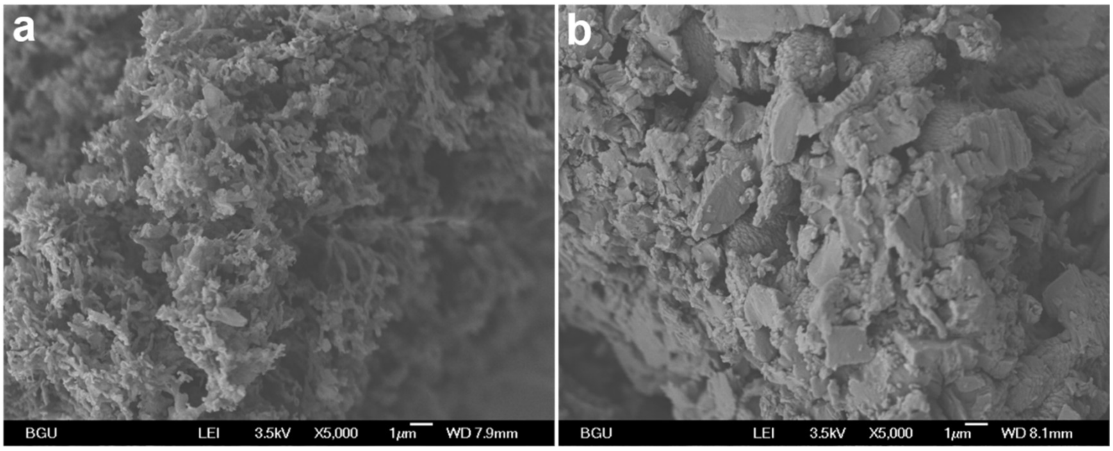

Supplementary Figure 17. SEM images of CN powders. a CN_U and **b** CN_D.

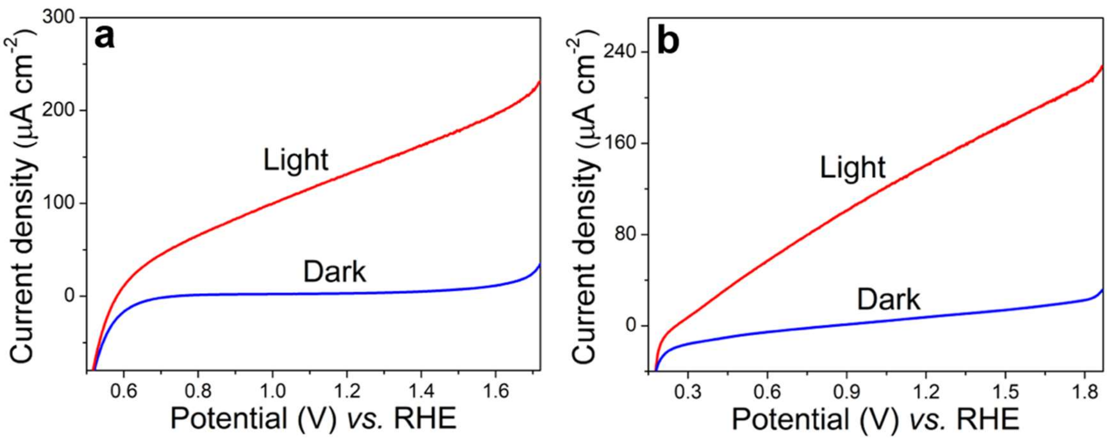

Supplementary Figure 18. LSV curves of different CN electrodes. **a** CN_U/FTO and **b** CN_D/FTO electrodes in 0.1 M KOH aqueous solution under 1-sun illumination (red), and in the dark (blue).

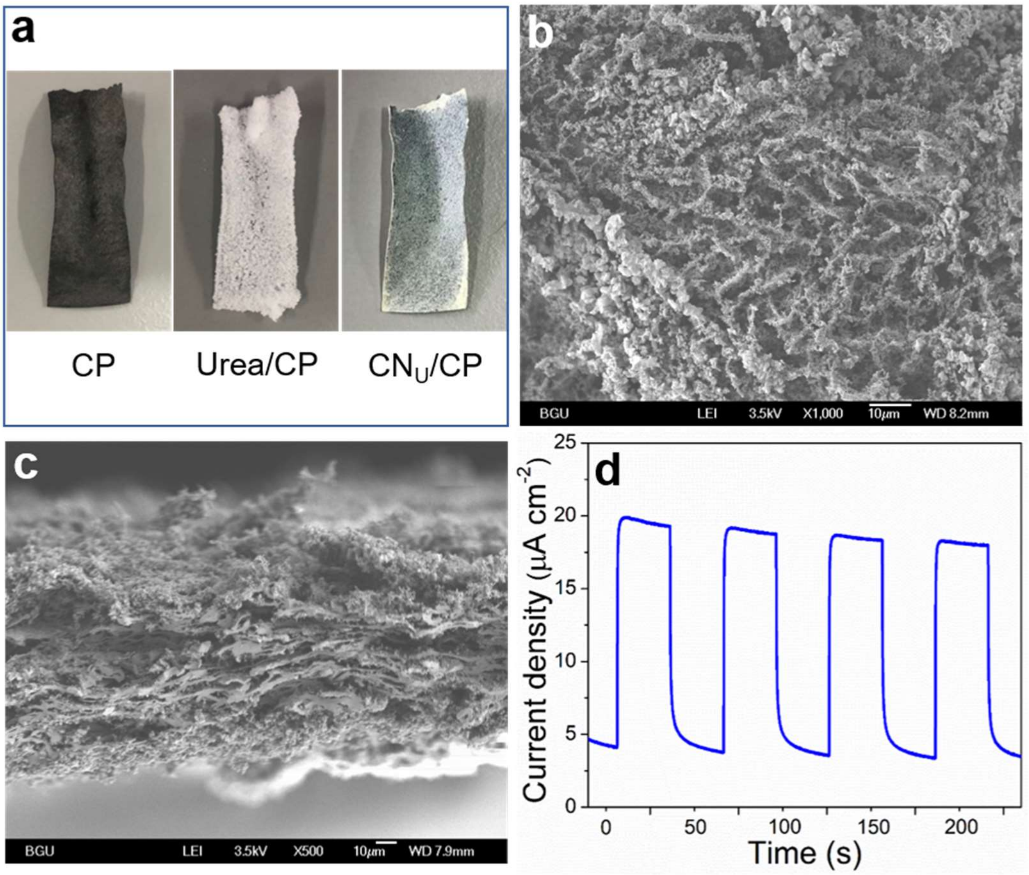

Supplementary Figure 19. CN_U film on carbon paper (CP). a Digital photos of CP, urea film on CP, and CN_U film on CP. **b** Top-view and **c** cross-sectional SEM images of CN_U film on CP. **d** Current density (chronoamperometry) of CN_U/CP electrode at 1.23 V *vs.* RHE in 0.1 M KOH upon 1-sun cycling illumination.

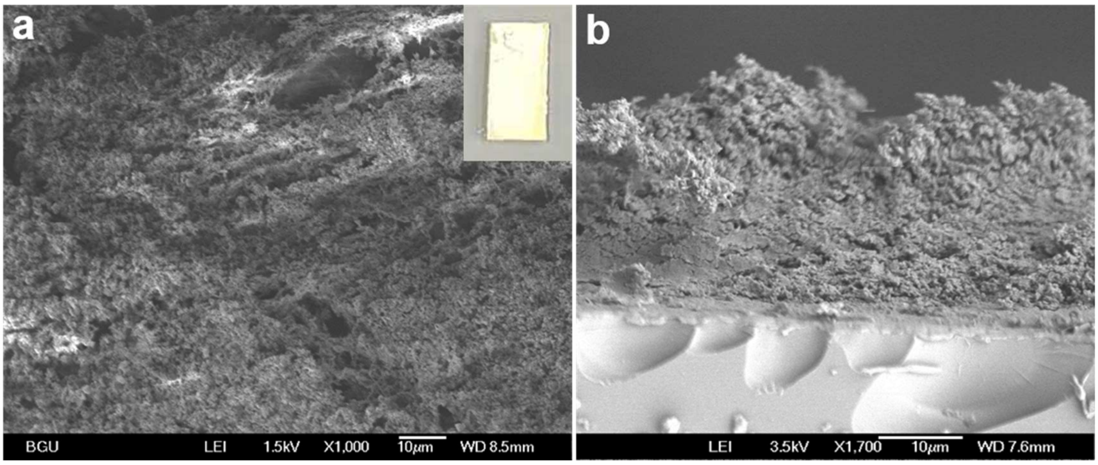

Supplementary Figure 20. SEM images of CN_U film on glass slide. a Top-view and **b** cross-sectional SEM images. (inset: a digital image of CN_U film on glass slide).

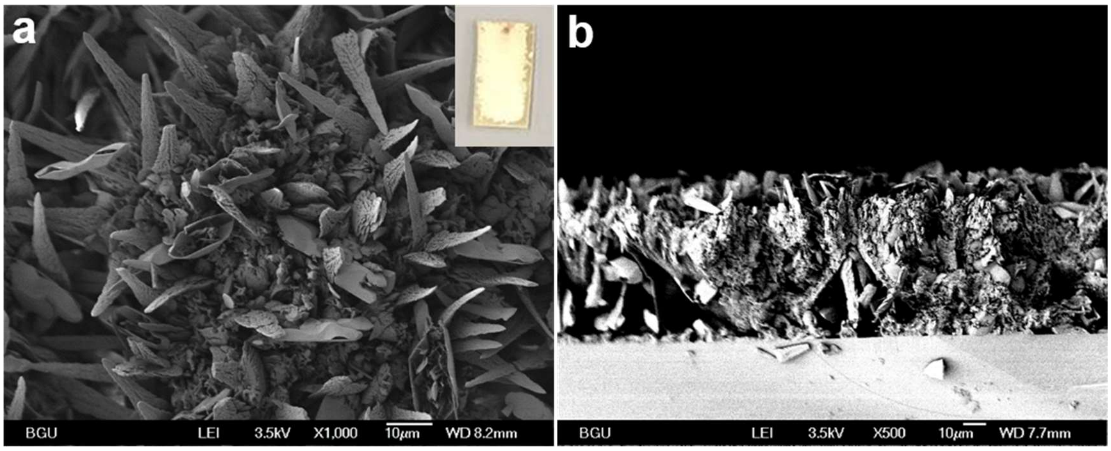

Supplementary Figure 21. SEM images of CN_D film on glass slide. a Top-view and **b** cross-sectional SEM images. (inset: a digital image of CN_D film on glass slide)

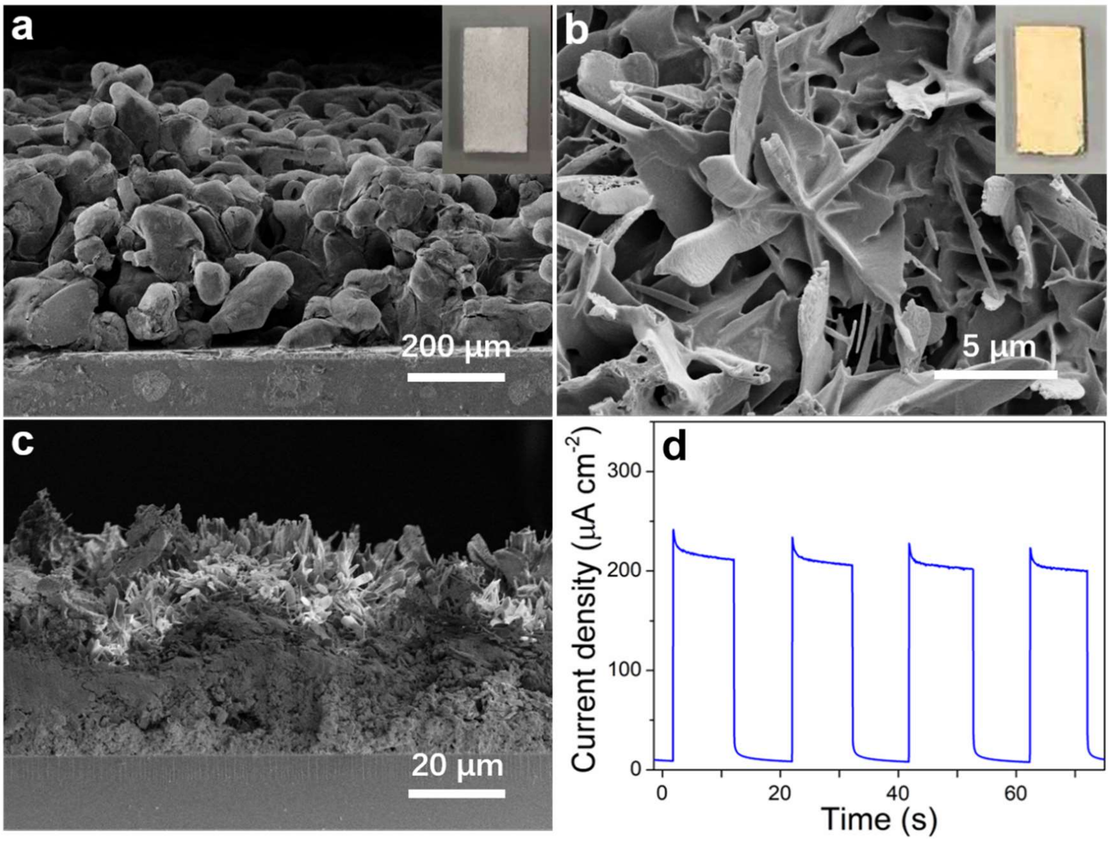

Supplementary Figure 22. CN film synthesized from NH_4SCN (NS). a Cross-sectional SEM image of NS film on FTO (inset: digital photo). **b** Top-view SEM image (inset: digital photo of CN_{NS}/FTO electrode) and **c** cross-sectional SEM image of CN_{NS}/FTO electrode. **d** Current density (chronoamperometry) of CN_{NS} electrode at 1.23 V *vs.* RHE in 0.1 M KOH upon 1-sun cycling illumination.

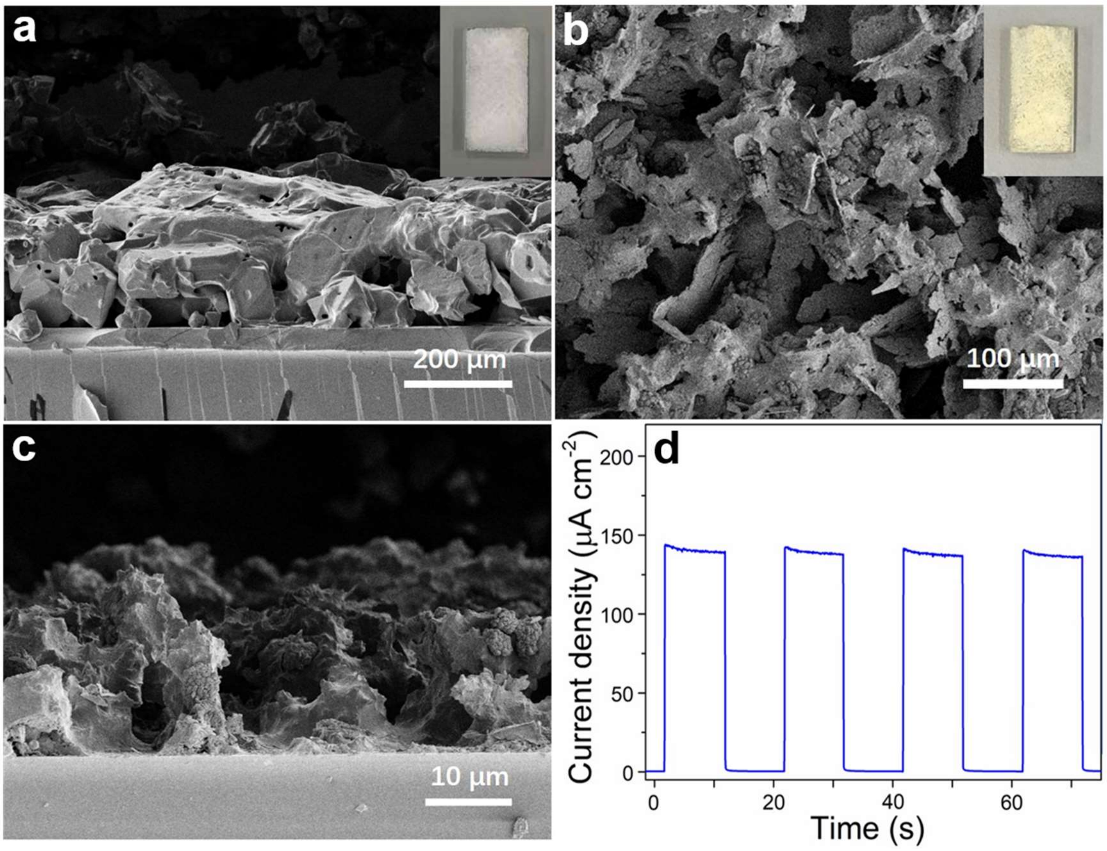

Supplementary Figure 23. CN film synthesized from guanidine carbonate (GC). a Cross-sectional SEM image of GC film on FTO (inset: digital photo). **b** Top-view SEM image (inset: digital photo of CN_{GC}/FTO electrode) and **c** cross-sectional SEM image of CN_{GC}/FTO electrode. **d** Current density (chronoamperometry) of CN_{GC} electrode at 1.23 V *vs.* RHE in 0.1 M KOH upon 1-sun cycling illumination.

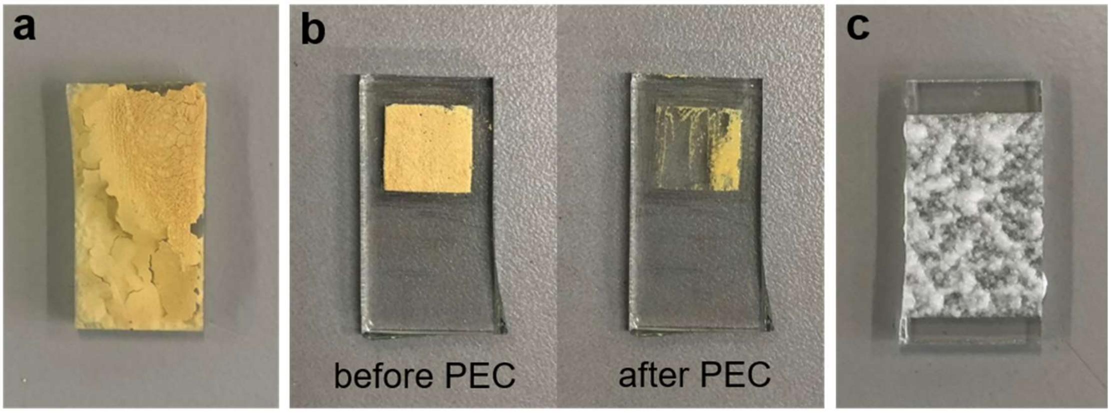

Supplementary Figure 24. Synthesis of CN_T film using different methods. a Calcination of thiourea powder that had been spread on FTO. **b** Drop-casting a CN_T powder slurry onto FTO, followed by calcination at 300 °C for 1 h (before (left) and after (right) a PEC experiment). **c** Application of a doctor blade technique, which does not result in a uniform film from thiourea as the precursor (here, shown using ethylene glycol as the binding solvent). All digital images taken of films on FTO-coated glass substrates.

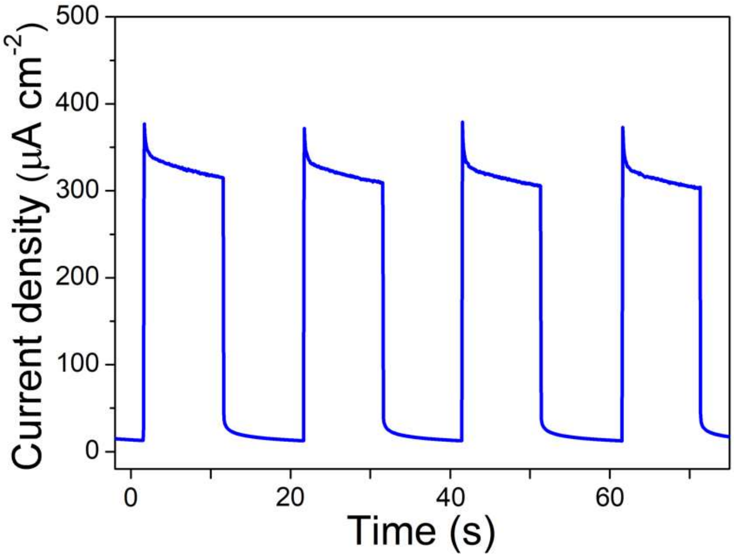

Supplementary Figure 25. Current density (chronoamperometry) of the CN_{TM} electrode calcined at 500 °C for 2 h. Measured at 1.23 V *vs.* RHE in 0.1 M KOH upon 1-sun cycling illumination.

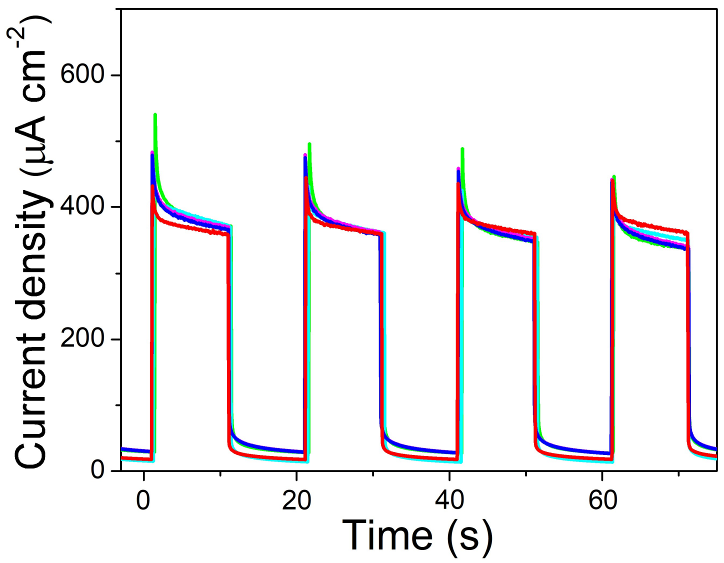

Supplementary Figure 26. Current density (chronoamperometry) of five different CN_{TM} electrodes. Five separate experimental procedures of electrode preparation and chronoamperometric characterization, measured at 1.23 V *vs.* RHE in 0.1 M KOH upon 1-sun cycling illumination are shown using blue, green, magenta, cyan, and red lines.

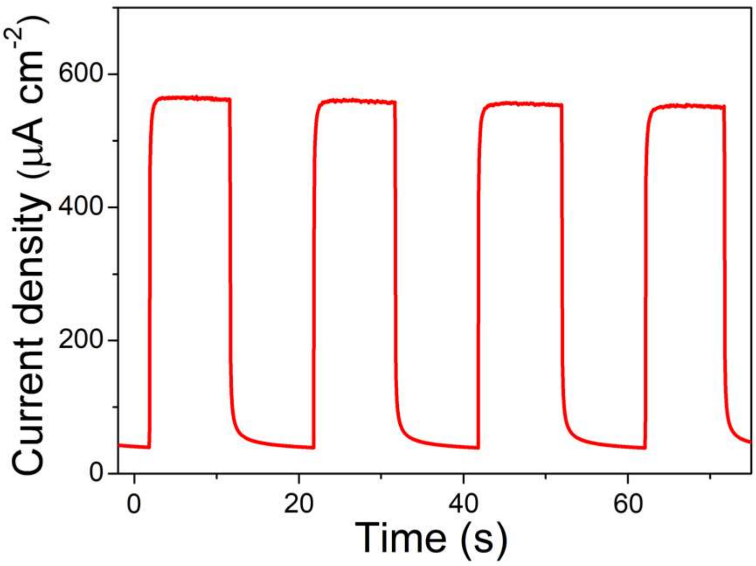

Supplementary Figure 27. Current density (chronoamperometry) of a CN_{TM} electrode. Measured at 1.23 V *vs*. RHE in 0.1 M KOH aqueous solution containing 10% (v/v) TEOA upon 1-sun cycling illumination.

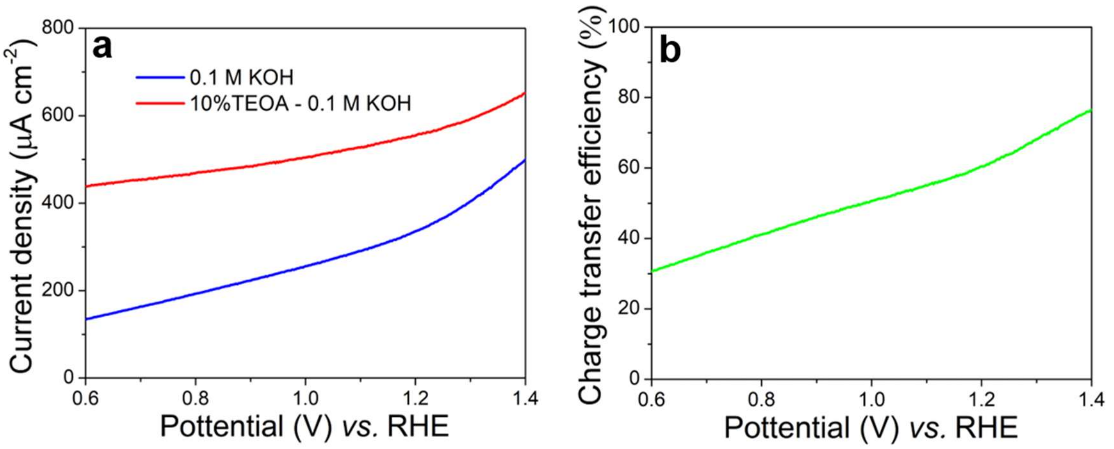

Supplementary Figure 28. Charge transfer efficiency of CN_{TM} electrode. a LSV curves of the CN_{TM} electrode in 0.1 M KOH with and without 10% (v/v) TEOA upon 1-sun illumination. **b** The charge transfer efficiency plot of the CN_{TM} electrode.

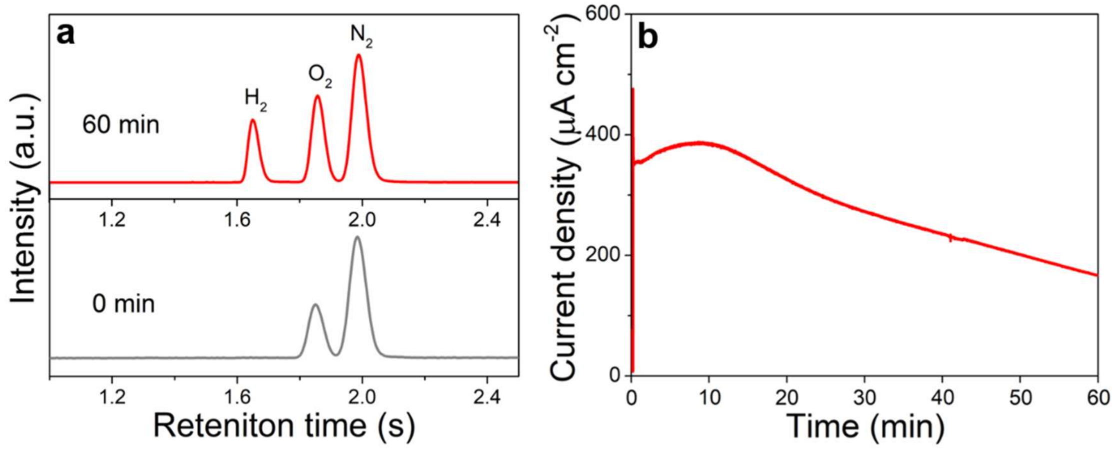

Supplementary Figure 29. PEC water splitting performance of CN_{TM} electrode under alkaline conditions. **a** Gas evolution detected by gas chromatography. Gray (bottom) curve: the GC signal before reaction; red (top) curve: the GC signal after 60 min reaction at 1.23 V *vs.* RHE in 0.1 M KOH upon 1-sun illumination. **b** Photocurrent density of CN_{TM} electrode at 1.23 V *vs.* RHE in 0.1 M KOH under 1-sun illumination measured during H_2 and O_2 evolution measurements.

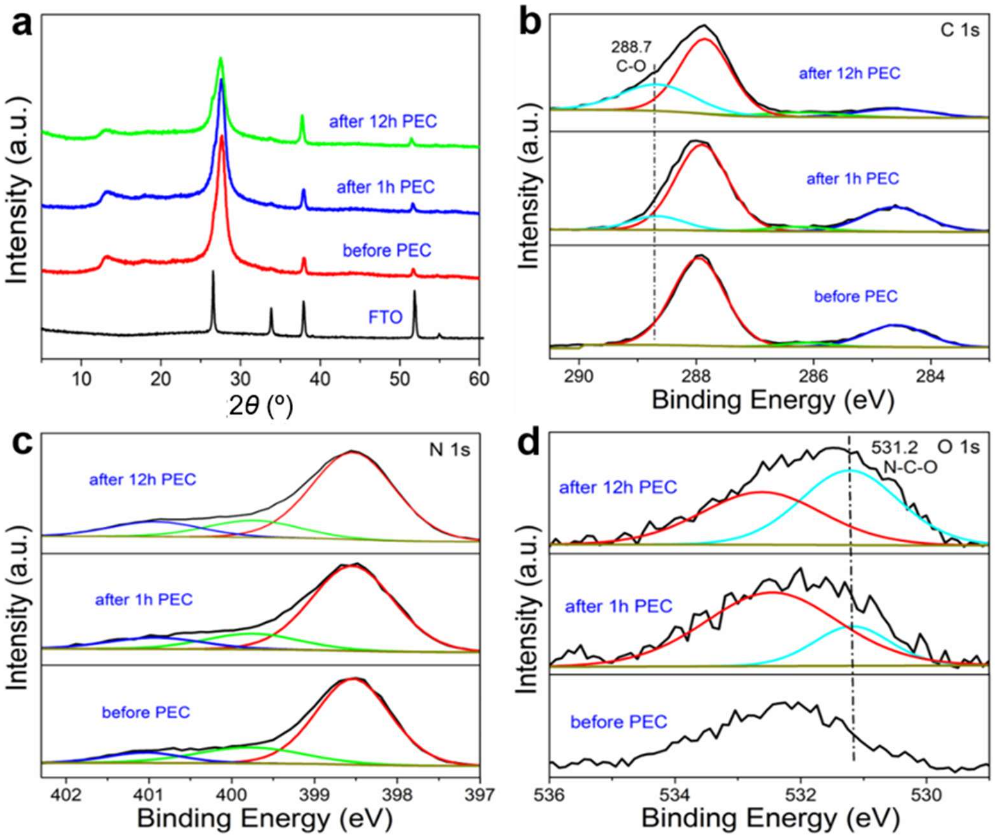

Supplementary Figure 30. Characterization of the CN_{TM} electrode after different PEC reaction times. a XRD patterns. XPS spectra of **b** C 1s, **c** N 1s, and **d** O 1s.

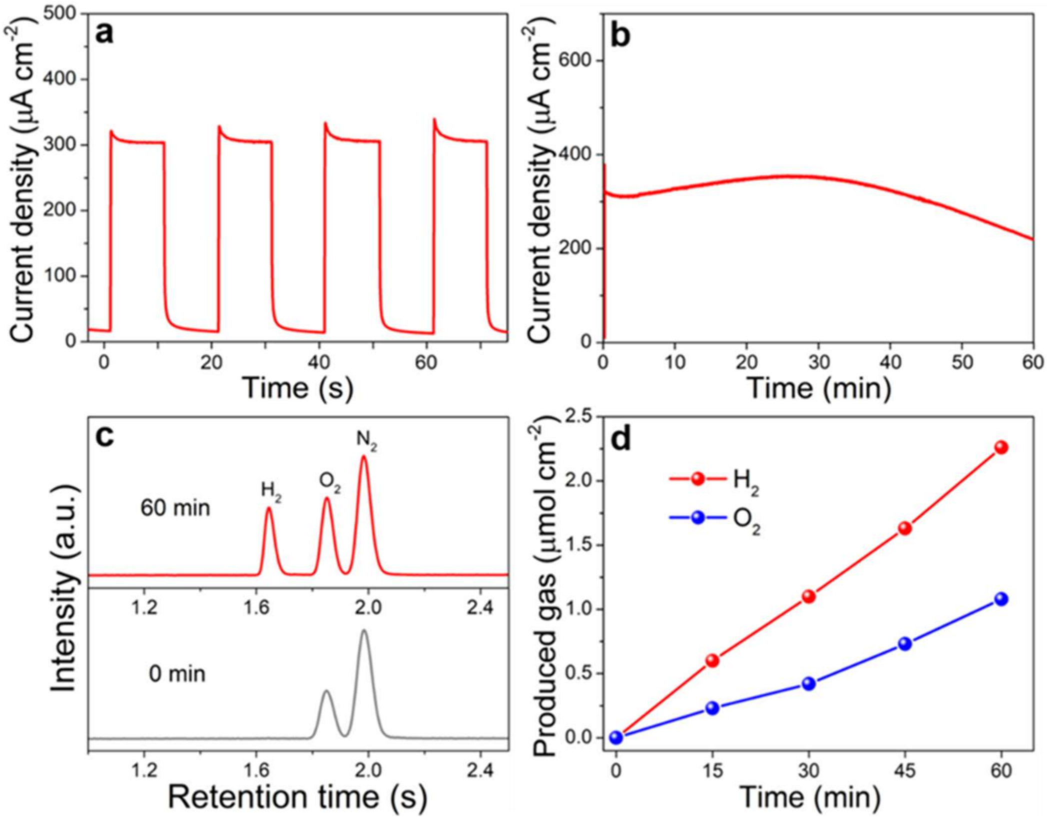

Supplementary Figure 31. PEC characterization of CN_{TM} electrode under neutral conditions. **a** Current density (chronoamperometry) upon 1-sun cycling illumination of CN_{TM} electrode at 1.23 V *vs.* RHE in 0.1 M Na_2SO_4 (pH = 7.02). **b** Photocurrent density of CN_{TM} electrode at 1.23 V *vs.* RHE in 0.1 M Na_2SO_4 under 1-sun illumination for H_2 and O_2 evolution measurements. **c** Gas evolution detected by gas chromatography. **d** Time-production plot of H_2 and O_2 measured by gas chromatography.

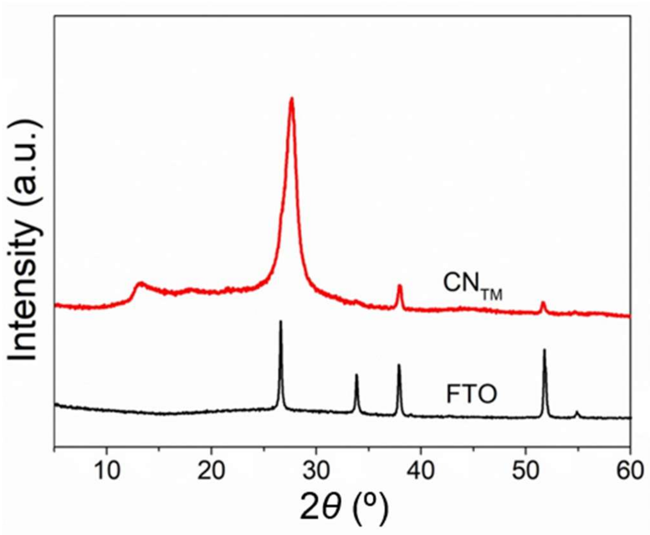

Supplementary Figure 32. XRD of CN_{TM} on FTO. Red pattern is CNTM on FTO and the black pattern is the reference clean FTO-coated glass spectrum.

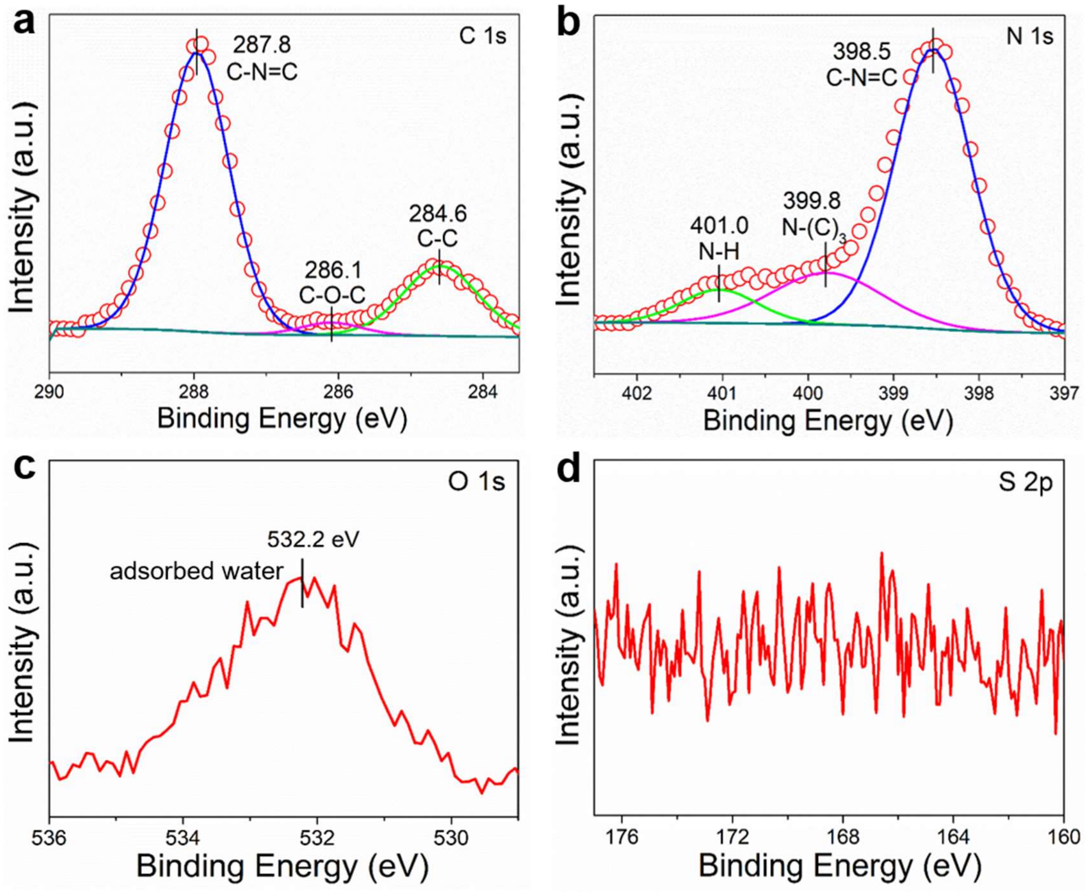

Supplementary Figure 33. XPS spectra of CN_{TM} electrode. a C 1s, **b** N 1s, **c** O 1s, and **d** S 2p.

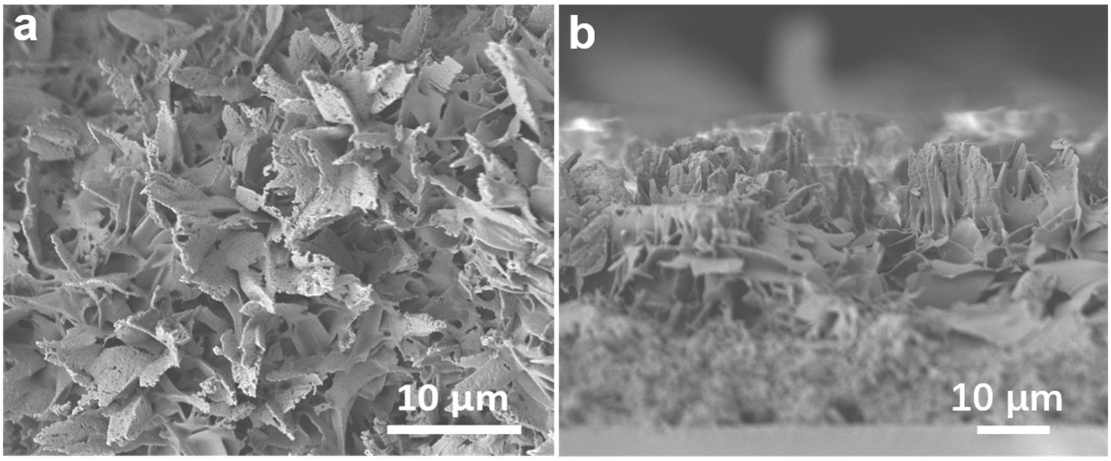

Supplementary Figure 34. SEM images of the CN_{TM} film on FTO. **a** Top-view and **b** cross-sectional SEM images.

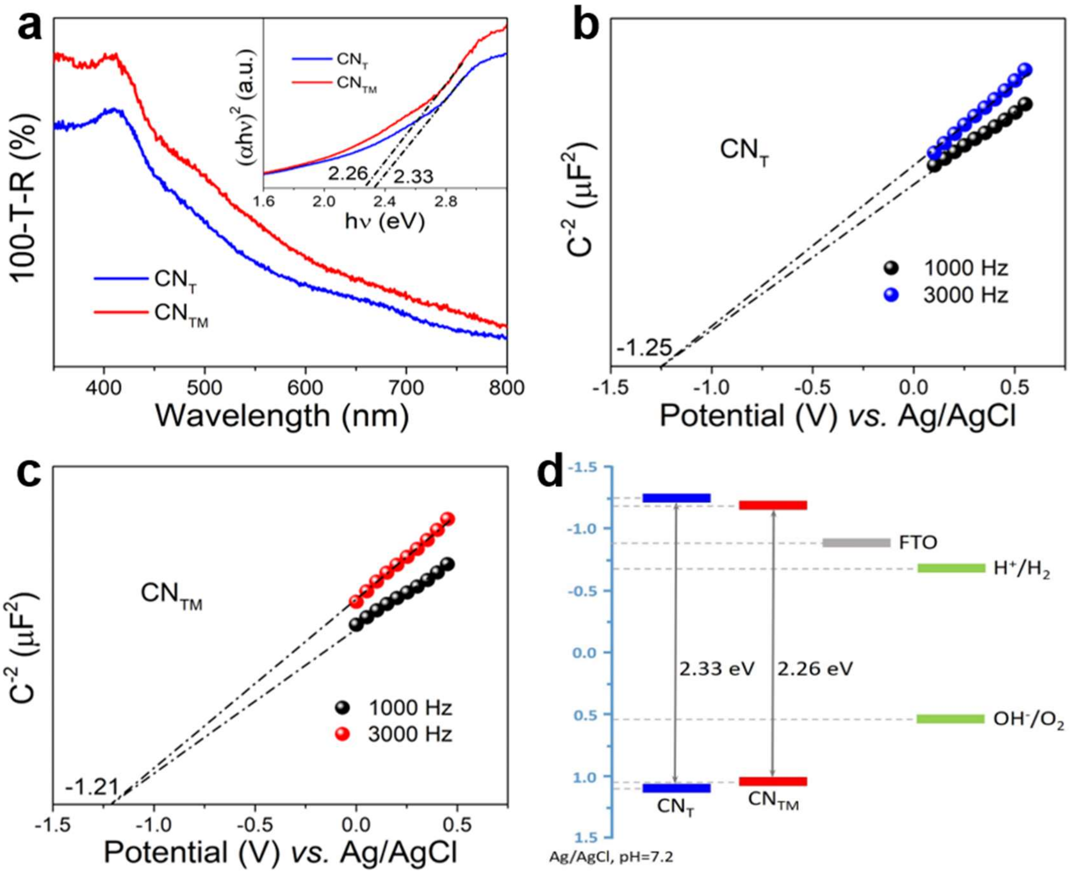

Supplementary Figure 35. Energy positions of CN_T and CN_{TM} electrodes. **a** UV-vis spectra of CN_T and CN_{TM} electrodes (inset: the corresponding Tauc plot analysis). Mott-Schottky plots of **b** CN_T and **c** CN_{TM}. **d** Energy levels diagram of CN_T and CN_{TM} electrodes with respect to water redox reactions (calculated from the Mott-Schottky plots for the valence band position, and the calculated band gap from the optical band gap obtained from the Tauc plot analysis).

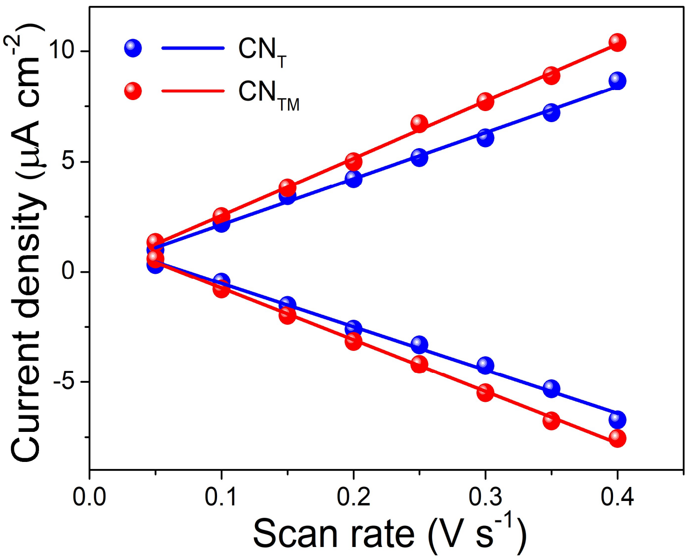

Supplementary Figure 36. Electrochemical active surface area (ECSA) measurements. Cathodic and anodic charging currents of CN_T (blue) and CN_{TM} (red) films on FTO measured at 0 V *vs.* Ag/AgCl as a function of scan rate.

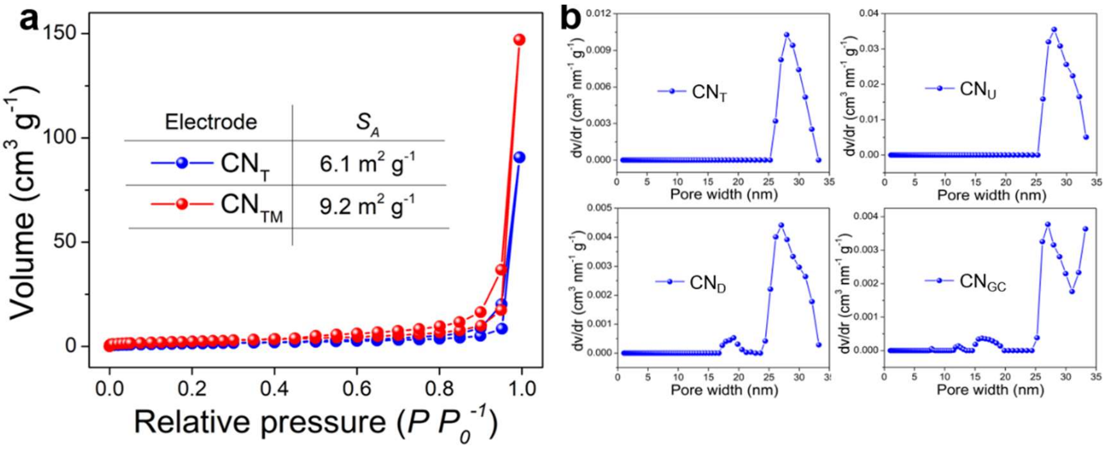

Supplementary Figure 37. N_2 adsorption-desorption measurements of different CN electrodes. **a** N_2 adsorption-desorption isotherms of CN_T and CN_{TM} electrodes with their respective specific surface area, calculated using the BET model ($S_A(CN_T)$ = 6.1 m^2 g^{-1}; $S_A(CN_{TM})$ = 9.2 m^2 g^{-1}). **b** Pore size distribution of different CN photoanodes prepared from thiourea (CN_T), urea (CN_U), dicyandiamide (CN_D), and guanidine carbonate (CN_{GC}), respectively. All four CN photoanodes show similar pore size distribution, and the calculated average pore diameters (in nm) are 28.03, 27.96, 27.05, and 27.03, respectively, calculated using non-localized density functional theory (NLDFT) from the nitrogen sorption measurements.

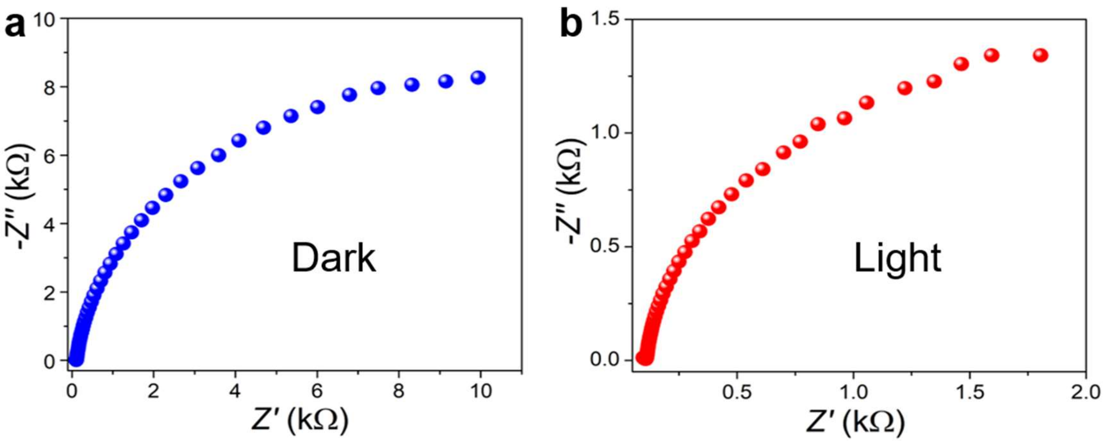

Supplementary Figure 38. Nyquist plots of the CN_{TM} electrode at 1.23 V *vs.* RHE. a Under dark condition; **b** Upon 1-sun illumination.

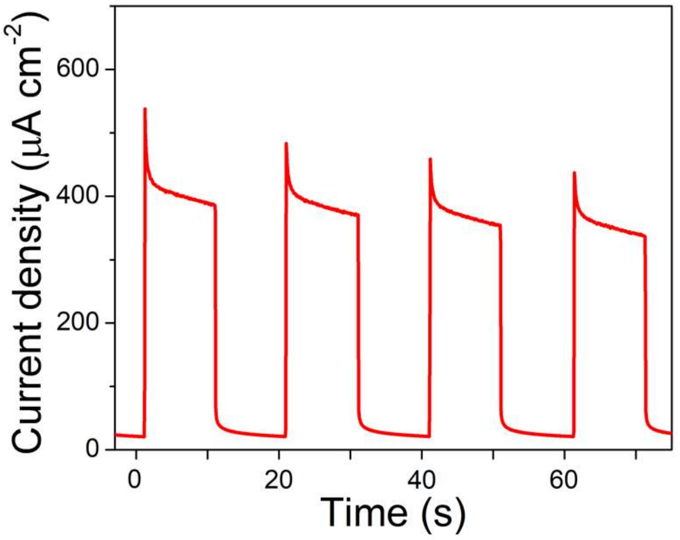

Supplementary Figure 39. Current density (chronoamperometry) of the CN_{TNS} electrode. Measured at 1.23 V *vs*. RHE in 0.1 M KOH upon 1-sun cycling illumination.

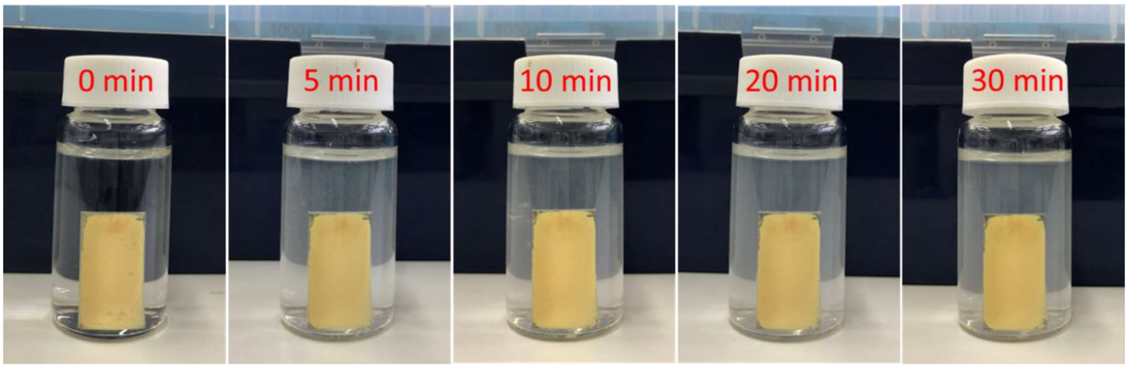

Supplementary Figure 40. Adhesion test of the CN_{TM} film on FTO by ultrasonication. Digital photos of CN_{TM} film on FTO slides after different time periods in water (0–30 min).

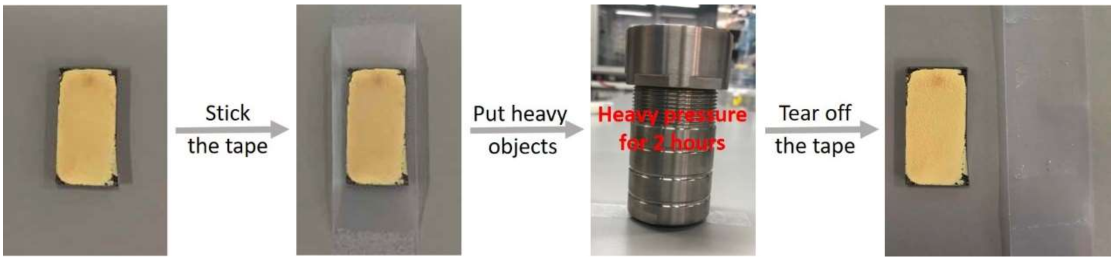

Supplementary Figure 41. Adhesion test of the CN_{TM} film on FTO by tape bonding. After ultrasonication, the CN_{TM} electrode underwent the shown process of tape bonding.

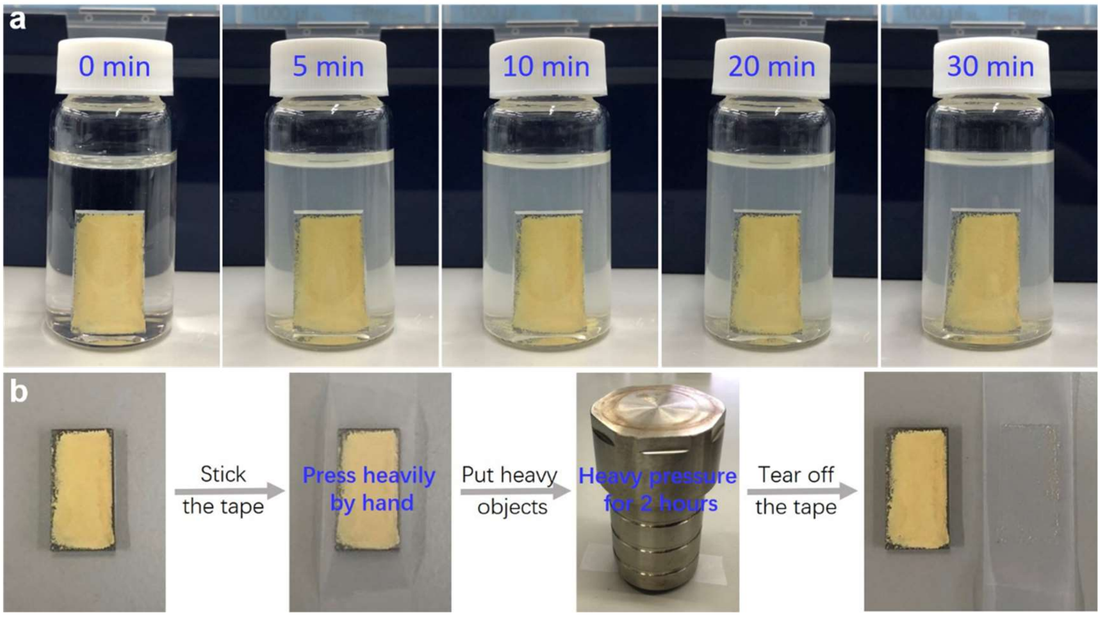

Supplementary Figure 42. Adhesion test of the CN_T film on FTO. a Digital photos of CN_T film on FTO slides after different ultrasonication times in water (0–30 min). **b** After ultrasonication, the CN_T electrode underwent the shown process of tape bonding.

Supplementary Tables

Supplementary Table 1. Summary of PEC performances of CN-based photoanodes.

Catalyst	Photocurrent (μA cm^{-2})	Potential *vs.* RHE (V)	Onset potential *vs.* RHE[a]	Electrolyte	Light intensity	Reference
CN_{TM}	353	1.23	0.32	0.1 M KOH	100 mW cm^{-2} AM 1.5	This work
	305	1.23	N/A	0.1 M Na_2SO_4	100 mW cm^{-2} AM 1.5	
	565	1.23	N/A	0.1 M KOH + 10% TEOA	100 mW cm^{-2} AM 1.5	
CN_T	266	1.23	0.38	0.1 M KOH	100 mW cm^{-2} AM 1.5	
	470	1.23	N/A	0.1 M KOH + 10% TEOA	100 mW cm^{-2} AM 1.5	
Porous CN/rGO film	124	1.23	0.3	0.1 M KOH	100 mW cm^{-2} AM 1.5	1
	272	1.23	N/A	0.1 M KOH + 10% TEOA	100 mW cm^{-2} AM 1.5	
P/B-layer-doping C_3N_4	150	1.23	N/A	0.1 M Na_2SO_4	300 W Xe lamp, AM 1.5	2
Phosphorylated CN	120	1.23	N/A	1.0 M NaOH	AM 1.5	3
Crystalline CN film	116	1.23	0.25	0.1 M KOH	100 mW cm^{-2} AM 1.5	4
	245	1.23	N/A	0.1 M KOH + 10% TEOA	100 mW cm^{-2} AM 1.5	
CN/rGO film	72	1.23	0.75	0.1 M KOH	100 mW cm^{-2} AM 1.5	5
	660	1.23	N/A	0.1 M KOH + 10% TEOA	100 mW cm^{-2} AM 1.5	
CN film	228.2	1.23	N/A (~ 0.65)	0.2 M Na_2SO_4	150 W Xe lamp, AM 1.5	6
CN	63	1.23	N/A	0.1 M Na_2SO_4	100 mW cm^{-2} AM 1.5	7
Boron-doped CN	103.2	1.23	N/A (~ 0.4)	0.1 M Na_2SO_4	100 mW cm^{-2} AM 1.5	8
3% Ni-CN	69.8	1.23	N/A	0.1 M KOH	100 mW cm^{-2} AM 1.5	9
Compact CN film	100	1.23	N/A	0.1 M Na_2SO_4 + 0.1 M Na_2SO_3 + 0.01 M Na_2S	100 mW cm^{-2} AM 1.5	10

CN	30.2	1.23	N/A	0.2 M Na_2SO_4	500 W Xe lamp	11
S-doped CN	60	1.23	N/A	0.1 M KOH	50 W (LED) white light	12

[a]Note: The values shown between parentheses (onset potential column) are estimated from the published LSV curves in the corresponding paper.

Supplementary Notes

Supplementary Note 1. The properties of CN materials are commonly affected by the calcination temperature. Herein, two additional electrodes (CN_{T450} and CN_{T550}) calcined at 450 °C and 550 °C were synthesized, respectively. Supplementary Figure 8a shows that the photocurrents of the two electrodes are still high, yet significantly lower than the values measured using CN_{T500} (red curve). XRD patterns (Supplementary Figure 8b) indicate that at 450 °C, graphitic-like networks are already formed, yet they are not complete. This observation is explained by the lack of sufficient thermal energy for complete thiourea condensation at 450 °C. Therefore, the lower and unstable photocurrent of CN_{T450} is attributed to the incomplete formation of CN. At 500 °C and 550 °C, the polymeric carbon nitride is well-formed, however, the CN_{T550} sample shows greater charge transfer resistance than the CN_{T500} one (Supplementary Figure 8c–d; blue vs. red in the Nyquist plots, respectively), especially under illumination. The higher resistance leads to the lower photocurrent of CN_{T550} relative to CN_{T500}.

Supplementary References

1. Peng, G., Qin, J., Volokh, M., Liu, C. & Shalom, M. Graphene oxide in carbon nitride: from easily processed precursors to a composite material with enhanced photoelectrochemical activity and long-term stability. *J. Mater. Chem. A* **7,** 11718-11723 (2019).
2. Zhang, J., Luan, P., Meng, Q., Wu, J., Li, Q., Zhang, X., Zhang, Y., O'dell, L., Raga, S. & Pringle, J. Unique Layer-Doping-Induced Regulation of Charge Behaviour in Metal-Free Carbon Nitride Photoanodes for Enhanced Performance. *ChemSusChem* **13,** 328-333 (2019).
3. Fang, Y., Li, X. & Wang, X. Phosphorylation of Polymeric Carbon Nitride Photoanodes with Increased Surface Valence Electrons for Solar Water Splitting. *ChemSusChem* **12,** 2605-2608 (2019).
4. Peng, G., Albero, J., Garcia, H. & Shalom, M. A Water-Splitting Carbon Nitride Photoelectrochemical Cell with Efficient Charge Separation and Remarkably Low Onset Potential. *Angew. Chem. Int. Ed.* **130,** 16033-16037 (2018).
5. Peng, G., Volokh, M., Tzadikov, J., Sun, J. & Shalom, M. Carbon Nitride/Reduced Graphene Oxide Film with Enhanced Electron Diffusion Length: An Efficient Photo-Electrochemical Cell for Hydrogen Generation. *Adv. Energy Mater.* **8,** 1800566 (2018).
6. Xiong, W., Chen, S., Huang, M., Wang, Z., Lu, Z. & Zhang, R. Q. Crystal-Face Tailored Graphitic Carbon Nitride Films for High-Performance Photoelectrochemical Cells. *ChemSusChem* **11,** 2497-2501 (2018).
7. Lv, X., Cao, M., Shi, W., Wang, M. & Shen, Y. A new strategy of preparing uniform graphitic carbon nitride films for photoelectrochemical application. *Carbon* **117,** 343-350 (2017).
8. Ruan, Q., Luo, W., Xie, J., Wang, Y., Liu, X., Bai, Z., Carmalt, C. J. & Tang, J. A nanojunction polymer photoelectrode for efficient charge transport and separation. *Angew. Chem. Int. Ed.* **56,** 8221-8225 (2017).
9. Zhang, W., Albero, J., Xi, L., Lange, K. M., Garcia, H., Wang, X. & Shalom, M. One-Pot Synthesis of Nickel-Modified Carbon Nitride Layers Toward Efficient Photoelectrochemical Cells. *ACS Appl. Mater. Interfaces* **9,** 32667-32677 (2017).
10. Bian, J., Xi, L., Huang, C., Lange, K. M., Zhang, R. Q. & Shalom, M. Efficiency enhancement of carbon nitride photoelectrochemical cells via tailored monomers design. *Adv. Energy Mater.* **6,** 1600263 (2016).
11. Liu, J., Wang, H., Chen, Z. P., Moehwald, H., Fiechter, S., van de Krol, R., Wen, L., Jiang, L. & Antonietti, M. Microcontact-Printing-Assisted Access of Graphitic Carbon Nitride Films with Favorable Textures toward Photoelectrochemical Application. *Adv. Mater.* **27,** 712-718 (2015).
12. Xu, J., Cao, S., Brenner, T., Yang, X., Yu, J., Antonietti, M. & Shalom, M. Supramolecular chemistry in molten sulfur: preorganization effects leading to marked enhancement of carbon nitride photoelectrochemistry. *Adv. Funct. Mater.* **25,** 6265-6271 (2015).
